# Graph neural networks for networked analysis of gestational diabetes risk factors: a multi method framework

**DOI:** 10.1038/s41598-026-57000-8

**Published:** 2026-06-09

**Authors:** Kranthi Kumar Lella, Shaik Abdul Nabi, Uddagiri Sirisha, G. Muni Nagamani, Poluru Eswaraiah, Chanumolu Kiran Kumar

**Affiliations:** 1 Manipal Institute of Technology, Manipal Academy of Higher Education, Manipal, India; 2 Department Of Computer Science and Engineering, AVN Institute of Engineering and Technology, Hyderabad, Telangana 501510 India; 3 Department Of Computer Science and Engineering, Prasad V Potluri Siddhartha Institute Of Technology, Kanuru, Vijayawada, Andhra Pradesh 520007 India; 4 Department of Computer Science and Engineering, Koneru Lakshmaiah Education Foundation Deemed to Be University, Green Fields, Vaddeswaram, Andhra Pradesh 522237 India; 5 Vignan’s Institute of Management and Technology for Women, Hyderabad, Medchal Dist, 501301 India

**Keywords:** Graph neural networks, Gestational diabetes mellitus, Temporal analysis, Explainable AI, Federated learning, Process, SDG 3, Computational biology and bioinformatics, Health care, Mathematics and computing

## Abstract

Gestational diabetes mellitus, often known as GDM, is a major health issue that causes complications for mothers and requires patient data prediction models that are complex and variable. The research in question makes use of graph-based learning in order to investigate the ways in which genetic, biochemical, and demographic elements interact in a variety of different contexts. Through the use of nodes to represent patients and lines to represent the things that they share in common, the framework illustrates how the aforementioned elements influence the likelihood of illness. Graph neural networks are utilized for the process, while BioBERT embeddings are utilized for the management of unstructured clinical notes. Graph neural networks are utilized for organized clinical notes. Because of this alignment, healthcare processes are placed in the context in which they should be, rather than being taken out of context while they are being carried out. The graph architecture used in BioBERT incorporates semantic patterns derived from medical information into a relational structure that illustrates the degree to which patients are similar to one another. After being evaluated on a substantial clinical dataset, the proposed method is able to make more accurate and readable predictions than the baseline models. The results of this study indicate that the utilization of graph architecture with both organized and unstructured data can assist in the discovery of novel approaches to the treatment of GDM that go beyond performance sets. According to the findings of the study, machine learning needs to be modified so that it can be used with healthcare applications.

## Introduction

Gestational diabetes mellitus is a major metabolic disorder occurring in pregnant individuals, and it has significant implications both for maternal health sets and fetal health sets. It characterizes itself with glucose intolerance during pregnancy. GDM increases the risks for complications such as macrosomia, preterm delivery, and long-term metabolic disorders in offspring. Today’s clinical methods^1–3^ for assessing GDM risk are mainly linear ones, based on isolated clinical factors and standard statistical approaches. These methods, though useful in controlled environments, cannot explain the intricate, interdependent, and dynamic nature of GDM risk factors such as demographic variables, genetic predispositions, lifestyle behaviors, and changes in clinical measurements over time. Recent breakthroughs in artificial intelligence, particularly in Graph Neural Networks (GNNs), are exciting directions for modeling this complexity. GNNs uniquely have the ability to model relational structures and multi-dimensional interactions within datasets & samples. This is quite critical in GDM analysis as risk factors often compose a network of interdependent variables with causal and temporal dependencies. Although promising in its concept^4–6^, currently, GNN has fewer applications in healthcare domains because most of the studies use static or homogeneous graphs that fail to exploit any information available in the graph structure and related edges, including heterogeneous relationship as well as dynamic temporal pattern. This paper introduces a novel framework for networked analysis of GDM risk factors using a suite of advanced GNN methodologies. This approach integrates feature aggregation via GraphSAGE, multi-typed relationships by R-GCN, and temporal evolution by TGAT. GAT is utilized for achieving explainability while ensuring the privacy-preserving federated learning for the secure deployment of models. This integration, therefore, not only improves the predictive accuracy but also offers more interpretable insights into factors contributing to GDM and bridges the gap between advanced machine learning techniques and practical healthcare needs.

Traditional algorithms cannot capture irregular, multi-relational patient data for gestational diabetes prediction. The model encodes demographic, clinical, and biochemical variable interactions using graph-based patient feature representations. This section contextualizes component roles in patient health trajectories rather than algorithmic descriptions. The basic model, Graph Neural Networks (GNNs), captures patient variable related interactions. Instead of algorithms, the argument compares message forwarding, neighborhood aggregation, and attention-driven early gestational diabetes risk detection options. These methods are specialized for correlating genetic markers, lifestyle patterns, and clinical data samples’ temporal fluctuations. With one pipeline, the method streamlines data preprocessing, graph building, representation learning, and prediction. Practical implementation approaches include incorporating patient nodes by merging clinical records and diagnostic codes and graph attention to prioritize clinically relevant elements. The section is now a problem-driven design story linking model structure to medical applications rather than an algorithm catalog.

Gestational diabetes mellitus is therefore a process singularly unique in its healthcare challenge. It is multidimensional and dynamic, by nature, to the process itself. Traditional prediction models are foundational but limit the capacity to represent such an intricate network of interdependent risk factors associated with GDM. They commonly resort to lonely variables or toy associations, which cannot comprehend causality, temporal flows and multi Variable inter-actions in process. This tendency has also led to another request for advanced methods that can accommodate and analyze heterogeneous data sets & samples because sources of different types of data are increasingly becoming available. Some examples of diverse sources include electronic health records, demographic surveys, and genetic data. The additional challenge imposed by privacy considerations, with patient data dispersed across different institutions, necessitates safe and decentralized analysis approaches. To mitigate these challenges, this paper develops a holistic framework based on Graph Neural Networks, employing six state-of-the-art methods tailored for GDM analysis. GraphSAGE offers scalable and robust embeddings of clinical and demographic data with missing values. It captures complex, multi-typed relationships, like causal and temporal dependencies between risk factors. TGAT incorporates the important temporal dynamics required to study the progression of GDM during pregnancy. GAT presents interpretable models by scoring attention to the most influential risk factors, hence supporting clinical decision-making. BioBERT domain-specific pretraining is on medical literature and for graph-based tasks, one can have robust embeddings at initialization. Finally, federated learning with differential privacy allows for the safe training of models on decentralized datasets, thereby addressing the privacy and regulatory concerns. All aspects of predictive accuracy, explainability, and privacy preservation have been improved upon in the proposed framework. It achieves superior performance metrics, such as an AUC of 0.9 for GDM prediction and over 80% agreement with domain experts in explainability assessments. This sets a new standard of work in GNN-based health care analytics and offers an approach scalable and interpretable in nature to address one of the most important public health concerns, opening up scope for more personalized and efficient GDM management.

## Contributions

The most important feature that we have included is the development and evaluation of a multi-method graph learning system for the prediction of gestational diabetes. Rather than listing algorithms in chronological sequence, this section makes use of research to provide solutions to tasks. The primary emphasis is placed on the ways in which combining approaches makes the handling of medical data anomalies more effective. In order to generate patient graphs, the framework takes into account demographic, clinical, and biochemical data. In the graph, each point represents a patient, and each edge indicates a characteristic or risk factor that is shared by all of the patients. This graphic is an example of something that traditional models do not display, such as patient groups that have similar DNA tendencies. Graph convolutional neural networks (GNNs) and attention-based GNNs have been proven to operate well together in order to identify local versus global dependencies; however, they are not effective in listing approaches. Third, repetitively combining images that we have learned alters the way that we carry out our daily activities. Clinicians are able to quantify risk and identify elements that play a role with a high degree of accuracy and ease of comprehension when they combine embeddings from a variety of models. In the final section of the article, it is demonstrated how the framework improves the accuracy of high-stakes medical predictions.

## Review of existing models for pregnancy diabetes analysis

During recent years, the incidence of gestational diabetes mellitus has reached an increasingly high level of concern, which has led to new waves of research about the early prediction and management of the disorder. This body of work was thus spread over the variety of approaches, from clinical studies up to more advanced machine learning models, and contributes to the development of improved strategies of diagnosis, treatment, and prevention of GDM. It ranges from clinical trials to biomarker studies, digital health technologies, and machine learning deployments. These studies included many of the aspects of GDM including pathophysiology, risk factors, and predictive models for early detection. Accordingly, the study of Moreno et al.^1^ provides much insight into the concerns of gestational weight gain information-seeking behavior of pregnant women, in particular, among immigrant Central American women in the United States; thus, both the social and behavioral understanding are essential due to their morphogenic risks that healthcare interventions might need to be framed about.

In like manner, Gulersen et al.^2^ discuss morbidity risks from early preterm birth in particular with respect to low-risk singleton pregnancies. It thus postulates that adverse pregnancy outcomes are connected with the factors of risks which culminate into GDM. Such studies thus help establish demographic, social, and medical determinants as pertinent to understanding and managing risks with GDM. Contributions to the physiological understanding of GDM include the study by Zampieri et al.^3^ on uterine artery indices and Genova et al.^4^ on the relationship between zinc status and insulin sensitivity, contributing to the clinical understanding of metabolic changes in GDM sets. These studies inform the medical community about the biological markers that could be used for early diagnosis and intervention sets. The works of Siemers et al.^5^ and Payot et al.^6^ report impairments in mitochondrial function and gene expression analyses, respectively at the molecular level; this should enlighten some mechanisms related to GDM and perhaps biomarkers at early stages of onset. Iteratively, Next, As shown in Table 1, In addition, Seifert et al.^7^ and Rüegg et al.^8^ explain the expression of some proteins like galectin-7 and how the perioperative care of women who undergo repair of fetal spina bifida affects GDM, representing how GDM interacts with other health conditions.

**Table 1:** Comparative analysis of existing methods.

Reference	Method		Main objectives		Findings		Limitations
Moreno et al.^1^	Survey study		Explore beliefs, concerns, and prenatal care advice regarding gestational weight gain among immigrant Central American pregnant women		Identified significant concerns regarding weight gain and information sources among this population, highlighting the need for tailored interventions		The study is region-specific and may not be generalized to other immigrant populations
Gulersen et al.^2^	Clinical study		Investigate maternal morbidity related to early preterm birth in low-risk singleton pregnancies		Found increased maternal morbidity with early preterm birth, emphasizing early monitoring and intervention		Limited to low-risk singleton pregnancies; results may not apply to high-risk cases
Zampieri et al.^3^	Diagnostic study		Study pulsatility and resistivity indices of uterine arteries in term pregnancies		Provided insights into uterine artery Doppler indices as potential markers for predicting adverse pregnancy outcomes		Small sample size and limited to term pregnancies
Genova et al.^4^	Biochemical study		Examine the relationship between zinc status and insulin resistance in gestational diabetes		Found that zinc status is associated with insulin resistance in GDM, suggesting it as a potential therapeutic target		Cross-sectional design; causality not established
Siemers et al.^5^	Molecular study		Investigate mitochondrial function and fatty acid uptake in human trophoblasts in gestational diabetes		Demonstrated that GDM impairs mitochondrial function and fatty acid uptake, impacting trophoblast function		In vitro study; may not fully replicate in vivo conditions
Payot et al.^6^	Biomarker study		Investigate potential early biomarkers for GDM through PTRPG and IGKV2D-28 expression		Identified specific biomarkers related to early GDM detection, offering potential diagnostic tools		Preliminary study; requires further validation in larger cohorts
Seifert et al.^7^	Molecular study		Investigate galectin-7 expression in placentas of women with GDM		Found altered galectin-7 expression in GDM placentas, suggesting it as a potential biomarker		Limited sample size and focus on a single biomarker
Rüegg et al.^8^	Clinical study		Examine the influence of perioperative management on GDM in women with fetal spina bifida repair		Revealed the importance of perioperative management in GDM outcomes, especially in complex cases like spina bifida repair		Narrow focus on a specific patient group; findings may not be generalizable
Hung et al.^9^	Experimental study		Study the effects of lysophosphatidylcholine on trophoblast function in GDM		Found that lysophosphatidylcholine impairs mitochondrial function, leading to trophoblast dysfunction in GDM		Limited to a specific lipid molecule, not considering broader metabolic factors
Filip et al.^10^	Clinical study		Examine the burden of deep vein thrombosis (DVT) during pregnancy and postpartum		Identified increased DVT risk during pregnancy and postpartum, particularly in high-risk groups		Focuses on DVT, not directly related to GDM but provides context for maternal health risks
Mandić Marković et al.^11^	Biochemical study		Evaluate biochemical markers for predicting pregnancy outcomes in GDM		Found several promising biomarkers for predicting GDM outcomes, aiding in early intervention		Requires validation in larger, diverse populations
Wang et al.^12^	Epidemiological study		Investigate the impact of beverage consumption during pregnancy on maternal and offspring outcomes		Found associations between beverage consumption and adverse outcomes, emphasizing dietary interventions		Cross-sectional design; cannot establish causal relationships
Lu et al.^13^	Digital health study		Review digital health technologies for blood glucose monitoring in GDM		Highlighted the potential of digital health and machine learning technologies for improving GDM management		Limited clinical validation of digital tools in real-world settings
Gomes Filho et al.^14^	Machine learning study		Develop a Bayesian network-based methodology for early GDM diagnosis		Found that Bayesian networks could effectively support early GDM diagnosis using clinical data		Limited to a single methodology; may not be applicable to all patient populations
Kolozali et al.^15^	Machine learning study		Combine medical background and wearable devices for early prediction of GDM biomarkers		Demonstrated the efficacy of combining traditional medical data with wearable devices for early GDM prediction		Pilot study with a small cohort, limiting generalizability
Marzouk et al.^16^	Predictive modeling study		Develop a predictive model for diabetes monitoring using IoT and machine learning		Created a secure, web-based personalized diabetes monitoring system using machine learning		Reliant on IoT infrastructure, which may not be widely available in all healthcare settings
Khan et al.^17^	Machine learning study		Use bio Inspired PSO to enhance neural network-based diabetes prediction		Applied particle swarm optimization to improve neural network performance for diabetes prediction		Limited to PSO and neural networks; other optimization techniques not considered
Saleh Al Reshan et al.^18^	Deep learning study		Develop an ensemble deep learning system for diabetes prediction		Found that ensemble deep learning models significantly improved diabetes prediction accuracy		Requires large datasets for training and may be computationally expensive
Linkon et al.^19^	Machine learning study		Evaluate feature transformation techniques for diabetes detection		Found that feature transformation techniques enhance the accuracy of machine learning models for diabetes detection		May require extensive preprocessing for large datasets
Alnowaiser et al.^20^	Machine learning study		Improve diabetes prediction using KNN imputed features and a tri-ensemble model		Demonstrated that KNN imputation combined with tri-ensemble models improved prediction accuracy		Dependent on feature imputation, which may introduce biases
Zhao et al.^21^	Machine learning study		Develop hypertension risk prediction models for diabetic patients		Created machine learning models that effectively predict hypertension risk in diabetic patients		Focus on hypertension may not directly apply to GDM-specific outcomes
Kurt et al.^22^	Deep learning study		Predict GDM using deep learning, Bayesian optimization, and traditional machine learning		Found that deep learning models outperformed traditional techniques in predicting GDM		The complexity of deep learning models requires significant computational resources
Ashisha et al.^23^	Machine learning study		Apply random oversampling techniques to improve diabetes classification		Found that random oversampling significantly improved classification performance for diabetes		May introduce overfitting if not carefully implemented
Feng et al.^24^	Machine learning study		Optimize diabetes classification using a machine learning-based framework		Demonstrated that an optimized machine learning framework enhances diabetes classification		Focused only on classification; does not account for other potential risk factors
Uddin et al.^25^	Machine learning study		Reduce false negatives in diabetes detection models		Developed a machine learning model specifically designed to reduce false negatives in diabetes detection		Limited generalizability due to focus on false negative reduction
Du et al.^26^	Machine learning study		Develop a visualization obesity risk prediction system		Created a machine learning-based system to predict obesity risk, potentially useful for GDM prediction		Focuses on obesity risk, which may not be directly transferable to GDM
Darsareh et al.^27^	Machine learning study		Identify risk factors for birth asphyxia using machine learning		Found that machine learning models can identify key risk factors for birth asphyxia, providing early intervention opportunities		Focus on birth asphyxia, not directly related to GDM
Wu et al.^28^	Machine learning study		Early prediction of GDM using maternal demographic and clinical factors		Developed a predictive model for GDM using demographic and clinical factors, improving early diagnosis		Relies on limited clinical variables; may not capture all relevant risk factors
Bai et al.^29^	Machine learning study		Predict large-for-gestational-age newborns in radiation-exposed women		Applied machine learning models to predict outcomes in women exposed to radiation before pregnancy		Limited to radiation-exposed women, affecting generalizability
Khadidos et al.^30^	Ensemble learning study		Create an ensemble framework for predicting maternal health risks		Developed an ensemble model that effectively predicts maternal health risks during pregnancy		May not work as well in populations with different health profiles
Du et al.^31^	Clinical study		Investigate maternal height as a risk factor for adverse GDM outcomes		Found that maternal height is an independent risk factor for adverse pregnancy outcomes in GDM		Limited to maternal height; other factors may need further exploration
Soria-Contreras et al.^32^	Longitudinal study		Investigate the lifetime impact of gestational diabetes on cognitive function		Demonstrated a potential long-term effect of gestational diabetes on cognitive function in midlife		Limited to a single cohort; larger studies are needed for confirmation
Yang et al.^33^	Machine learning study		Analyze neurodevelopmental delay in offspring using maternal risk factors		Used machine learning to identify maternal and perinatal factors associated with neurodevelopmental delay in offspring		Limited by the quality of population data used in the analysis
Doğru et al.^34^	Ensemble learning study		Hybrid super ensemble model for early-stage diabetes risk prediction		Developed a hybrid ensemble model that improved early-stage diabetes risk prediction		Dependent on complex model structures, making it computationally expensive
Xu et al.^35^	Epidemiological study		Investigate dietary inflammatory index as a predictor of prediabetes		Found that a higher dietary inflammatory index correlates with a greater risk of prediabetes in women with prior GDM		Focused on diet, which may not account for all relevant metabolic factors
Rustam et al.^36^	Machine learning study		Enhanced diabetes detection using ensemble feature engineering		Developed an ensemble feature engineering approach to improve diabetes detection accuracy, significantly enhancing model performance		The approach may require large amounts of data to effectively train and may be sensitive to the quality of input features
Ordoñez-Guillen et al.^37^	Machine learning study		Classify Type 2 diabetes mellitus subtypes using machine learning algorithms		Identified distinct subtypes of Type 2 diabetes, improving prediction and personalized treatment		Focused only on Type 2 diabetes subtypes, which may not apply to GDM diagnosis and management
Salvatori et al.^38^	Data-driven study		Use cluster analysis to identify and validate GDM subgroups		Found that data-driven cluster analysis could identify distinct subgroups within GDM patients, potentially improving personalized treatment strategies		Limited to cluster analysis; may miss other subgroups that do not fit into the identified categories
Naito et al.^39^	Machine learning study		Investigate associations between environmental factors and cardiometabolic diseases		Revealed heterogeneous associations between environmental factors and cardiometabolic diseases, using polygenic risk scores		Focused on cardiometabolic diseases, which may not directly apply to GDM, and relied on polygenic risk scores
Belaghi et al.^40^	Predictive modeling study		Predict preterm birth risk in multiparous women using logistic regression and machine learning		Demonstrated that both logistic regression and machine learning models could effectively predict preterm birth in multiparous women		Results may not be generalizable to all populations, particularly those with single pregnancies or different risk factors
Solomon et al.^41^	Review study		Review the role of machine learning for multifactorial genetic disorder prediction		Extensive review of machine learning applications in predicting multifactorial genetic disorders, including diabetes		Review-based; lacks original data or novel insights from new models or approaches
Long et al.^42^	Comparative study		Compare machine learning algorithms for predicting microalbuminuria risk		Showed that machine learning algorithms could be effectively applied to predict microalbuminuria risk, an important complication of diabetes		Limited to microalbuminuria and may not address other diabetic complications like GDM
Aghajanian et al.^43^	Predictive modeling study		Predict post-delivery hemoglobin levels with machine learning algorithms		Found that machine learning models can accurately predict post-delivery hemoglobin levels, potentially guiding clinical interventions		Results may not directly apply to GDM, as it focuses on postpartum hemoglobin levels
Kokori et al.^44^	Narrative review		Review the role of machine learning algorithms in the detection of gestational diabetes		Reviewed current evidence on the application of machine learning algorithms for GDM detection, highlighting various techniques		Limited by the narrative review format; lacks original experimental data and may omit certain approaches
Kaya et al.^45^	Machine learning study		Early prediction of gestational diabetes mellitus using machine learning models		Showed that machine learning models can predict GDM at early stages with high accuracy, potentially improving early intervention		Relied on a specific set of models; other predictive approaches may be overlooked
Kang et al.^46^	Machine learning study		Predict GDM in Asian women using machine learning algorithms		Demonstrated the efficacy of machine learning algorithms in predicting GDM in Asian women, accounting for ethnic-specific risk factors		Focused solely on Asian women, limiting the applicability of the results to other ethnic groups
Cubillos et al.^47^	Machine learning study		Develop models to predict GDM risk in the first half of pregnancy		Developed predictive models for GDM risk early in pregnancy, allowing for early interventions		Limited by the dataset and may not account for other complex, unmeasured factors in GDM development
Chan et al.^48^	Machine learning study		Use elemental contents in fingernails for early prediction of GDM		Found that machine learning models using elemental contents in fingernails could predict GDM early, offering a non Invasive diagnostic tool		The technique is novel but requires validation across larger and more diverse populations
Li et al.^49^	Machine learning study		Use machine learning to predict GDM in the first trimester		Demonstrated that machine learning algorithms can predict GDM in the first trimester with high accuracy, aiding early intervention strategies		The first trimester prediction model may require additional clinical validation in broader populations
Nuthakki et al.^50^	Machine learning study		Early detection of diabetes risk factors for improved health management		Created a machine learning-based system for early detection of diabetes risk factors, aimed at improving overall health management		Focused primarily on early detection; may require integration with existing clinical workflows for effective use

Iteratively, Next, based on Table 1, In addition, Seifert et al.^7^ and Rüegg et al.^8^ report the protein expression, for example, galectin-7, of perioperative management in female patients undergoing fetal spina bifida repair, hence demonstrating how GDM might interact with other medical conditions. Machine learning approaches toward predicting GDM have lately become a central theme in the literature. Several publications have appeared using artificial intelligence for predictive modeling. For instance, Hung et al.^9^ studied the effects of lysophosphatidylcholine on mitochondrial function in GDM. Such research contributes to a trend of combining state-of-the-art computational approaches with biological data towards improving risk prediction. A further integration of data from various sources, such as wearable devices and clinical records, which has been considered a promising area for early diagnosis. For example, Gomes Filho et al.^14^ used Bayesian networks for the prediction of GDM in order to illustrate how a combination of more traditional machine learning techniques and newer computational tools can improve the prediction accuracy. In this context, Kolozali et al.^15^ focus on early prediction of biomarkers for GDM by background medical information combined with wearable devices, and once more, data integration plays an important role in predictive analytics. Marzouk et al.^16^ as well as Khan et al.^17^ used large datasets through machine learning, which helps in the upgrading of prediction systems of diabetes and the decrease in false-negative cases. Their findings signify the tremendous potential of employing machine learning in fine-tuning risk evaluation. In order to upgrade the predictive efficacy, ensemble models as well as feature transformation techniques proved to be useful according to Saleh Al Reshan et al.^18^ and Linkon et al.^19^. These studies are further complemented by the work of Alnowaiser^20^, Zhao et al.^21^, and Kurt et al.^22^, where the idea of using several machine learning algorithms for enhanced predictions especially in personalized healthcare has been a subject of discussion. This includes, for instance, tri-ensemble and KNN imputed features applied by Alnowaiser, as these emphasize more the scope of hybrid models to provide better prediction regarding diabetes risks in various populations.

Another important contribution to the field is the use of digital health technologies. Lu et al.^13^ discuss how digital health and machine learning technologies are used for tracking blood glucose levels in pregnant women, emphasizing the role of real-time, data-driven health management tools. The integration of digital solutions and machine learning models brings new chances to manage GDM more in line with the person’s characteristics and proactivity. For both patients and providers, these possibilities are new. Other relevant studies include the work done by Uddin et al.^25^ and Du et al.^26^, that further supports the growing body of works regarding applications of machine learning into maternal health to predict risks such as obesity and birth asphyxia. These studies, though not limited only to GDM, provide insight into how machine learning can be applied to cross-sectional studies in terms of maternal and fetal health outcomes. As the studies further explore how machine learning can be implemented to determine the predictive capabilities associated with GDM, integration of clinical risk factors through algorithms of machine learning and adverse maternal outcomes is achieved in those studies. Bai et al.^29^ and Khadidos et al.^30^ have made predictions on risk issues regarding maternal health through the application of ensemble frameworks. Similarly, Du et al.^31^ and Soria-Contreras et al.^32^ also study risk factors by maternal height and long-term cognitive effects of GDM. Moreover, Yang et al.^33^, with their inclusion of population data and neurodevelopmental outcomes, reflects an expanding sense of awareness regarding a greater spectrum of GDM impact outside pregnancy condition sets. The work by Doğru et al.^34^ and Xu et al.^35^ points out the diet and inflammation role in predictive models of prediabetes and GDM, that is, it emphasizes the lifestyle factors in the design of predictive models. It is also novel in work by Rustam et al.^36^ and Ordoñez-Guillen et al.^37^, who employ ensemble feature engineering techniques to improve diabetes prediction due to better data representation. Such studies are indicative of the promise that personalized, lifestyle-oriented risk models may hold, that can be applied in real-time clinical environments for diabetes management. On the other hand, Salvatori et al.^38^ and Naito et al.^39^ focused more on cluster analysis and polygenic risk score use for the identification of subgroups within GDM, which provides more insight into the heterogeneity of the condition. This is further strengthened by the work of Belaghi et al.^40^, where logistic regression and machine learning are applied to add complexity to obstetric prediction models for a preterm birth prediction. The studies by Solomon et al.^41^ and Long et al.^42^ also depict integration in using multifactorial data to make predictions of both maternal as well as offspring outcomes toward advanced applications of machine learning in multifactorial disorder prediction.

Recent developments regarding machine learning-based GDM prediction methods are being studied. These studies, conducted by Kaya et al.^45^, Kang et al.^46^, and Cubillos et al.^47^, involve the addition of early risk factors especially in ethnic groups. New biomarkers and environmental factors are introduced in these studies, which help in predicting GDM, based on the basic research done prior to these studies. Li et al.^49^ and Nuthakki et al.^50^ show how machine learning algorithms can be used in the first trimester to improve early detection of GDM, which will be essential for better health outcomes in both Mothers and their babies.

In brief, this series of studies explores the multifaceted nature of GDM from biological mechanisms and biomarkers to machine learning models and digital health solutions. Convergence of clinical data, machine learning, and digital health technologies promises to revolutionize the management of GDM. In the near future, more accurate, real-time tools for early detection and personalized intervention sets will be offered to clinicians. Integration of diverse data sources and continued development of machine learning models will continue to improve the prediction and management of GDM, hence leading to better outcomes for both Mothers and their children in different scenarios.

## Proposed model

**Fig. 1:**
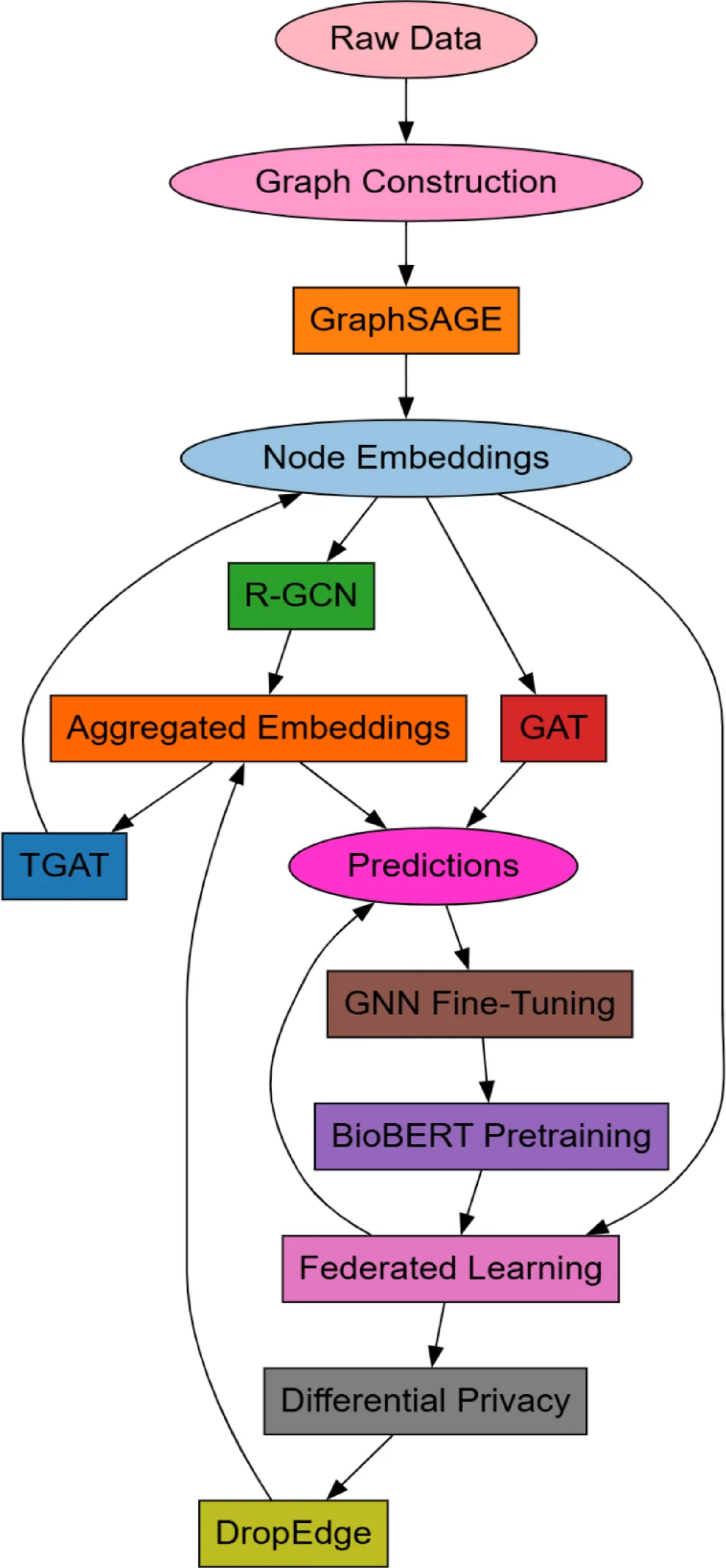
Model architecture for the proposed analysis process.

**Fig. 2:**
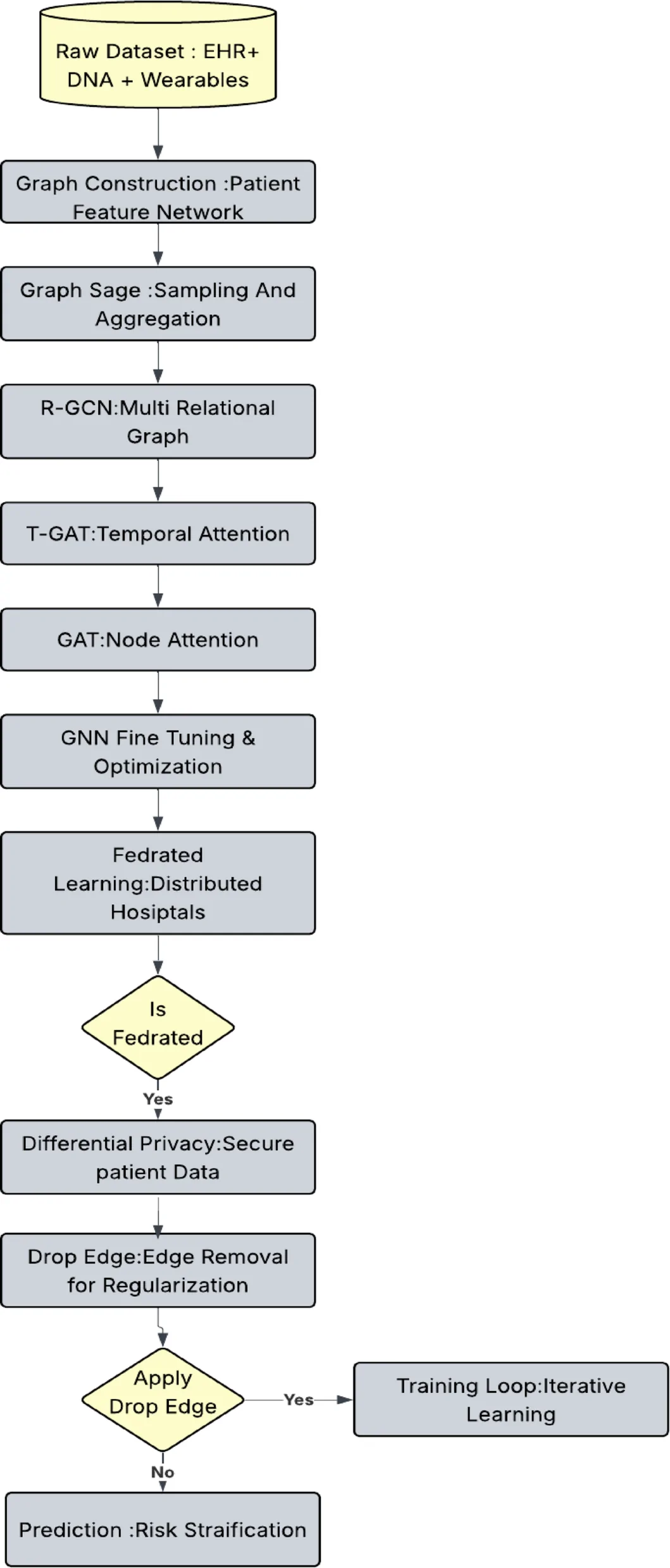
Overall flow of the proposed analysis process.

This section presents design of an iterative Multi Method Framework that uses Graph Neural Networks for Networked Analysis of Gestational Diabetes Risk Factors, to overcome issues of low efficiency & high complexity which are present in existing methods. Initially, as in Fig. 1, The proposed framework integrates GraphSAGE, Relational Graph Convolutional Network (R-GCN), and Temporal Graph Attention Network (TGAT) as core components for analyzing gestational diabetes mellitus (GDM) risk factors. Together, these methods allow for robust learning from complex, heterogeneous, and temporally dynamic datasets and samples. Each method is used to target different challenges posed when modeling GDM risk factors—scalability, relational heterogeneity, and temporal evolutions. GraphSAGE acts as the base graph representation, aggregating information from its neighboring nodes to create the node embeddings. This approach solves the problem of scalability in large graphs, particularly in clinical datasets where data incompleteness and dynamic additions (for example, new patients) are common for the process. Mathematically, for a node ‘v’, the embedding hv’k at the k-th layer is computed via Eq. 1,

1 $$h{v}{\prime}k = \sigma \left(Wk \cdot AGGREGATEk\left(\left\{h{u}{\prime}\left(k-1\right): u \in N\left(v\right)\right\}\right)\right)$$

where, Wk is the weight matrix for the k-th layer, σ is an activation function (ReLU), and N(v) denotes the neighbours of ‘v’ in the process. The aggregation function like mean or LSTM-based pooling is represented as AGGREGATE in the process. Embedding at the last layer catches both the local as well as global structural properties. This methodology is selected based on its ability to induce learning, meaning the model can generalize to unseen nodes, a critical requirement in datasets that have dynamic additions of patients. Iteratively, Next, as illustrated in Fig. 2, Relational Graph Convolutional Network (R-GCN) is incorporated to capture the multi-relational structure inherent in GDM risk factors, including the relationship between clinical, demographic, and environmental variables in the process.

For a given node ‘*v*’, the *k*-th layer embedding is computed via Eq. 2,

2 $$h{v}{\prime}k = \sigma \left(\sum_{r\in R}\sum_{u\in Nr\left(v\right)} \left(c(r,v)\right)\cdot W{r}{\prime}k h{u}{\prime}\left(k-1\right)+ W{0}{\prime}k h{v}{\prime}\left(k-1\right)\right)$$

where, R is a set of relation types. Nr(v) represents neighbors of ‘v’ for a given relation ‘r’. c(r,v) is a normalization constant, Wr’k and W0’k are weight matrices representing weight of relation-specific terms as well as self-loop terms. This formulation is the relation-aware one which allows for the differentiation between dependency kinds like causal and temporal during this process. The R-GCN architecture completes GraphSAGE by providing heterogeneity to the graph representation, thereby achieving a better understanding of the multi-typed relationships of the data samples. Successively, Next, following Fig. 2, Temporal Graph Attention Network (TGAT) further extends static graph analysis by GraphSAGE and R-GCN into temporal dynamics. In this, TGAT employs time-encoded embeddings that represent the time evolution of node features by reflecting the change in GDM risk factors over trimesters. For a node ‘v’ at timestamp t, the embedding is computed via Eq. 3,

3 $$hv\left(t\right)= ATTENTION\left(\left\{hu\left({t}{\prime}\right)+ \varphi \left(t-{t}{\prime}\right): u \in N\left(v\right), {t}{\prime}\le t\right\}\right)$$

where, φ(t − t′) is a timestamp encoding function, and ATTENTION represents an attention mechanism that assigns weights to neighboring nodes based on temporal relevance sets. The timestamp encoding function can be defined via Eq. 4.

4 $$\varphi \left(t-{t}{\prime}\right)= sin\left(\frac{t-{t}{\prime}}{\tau }\right)$$

where, τ is a scaling parameter to adjust the granularity of temporal encoding process. By aggregating time-weighted embeddings, TGAT enables the model to predict future states of GDM risk factors. The overall objective function for training the combined framework is defined via Eq. 5,

5 $$L = \left(\frac{1}{\left|V\right|}\right) \sum_{v\in V} \left[{\ell}\left(hv, yv\right)+ \lambda \sum_{k=1}^{K} \left|\left|Wk\right|\right|{F}^{2}\right]$$

where, ℓ(hv, yv) denotes the prediction loss for node ‘v’ (for example, cross-entropy for classification or mean squared error for regression), and λ||Wk||F is an L2 regularization term to prevent overfitting operations. The final node representation is formed by concatenating the embeddings generated by GraphSAGE, R-GCN, and TGAT, ensuring that both relational and temporal features are captured by the process. This combined design ensures that the framework exploits the strengths in the following: scalability of GraphSAGE, relational modeling of R-GCN, and TGAT’s temporal adaptability levels. Integrating these methods into the system enables it to solve special problems posed by GDM data and thus sets for high accuracy predictivity in the framework and puts forth for interpretability. The use of advanced architectures such as GNN in a sophisticated way brings the precision of risk predictions, which yet enables clinical decision making upon a holistic inspection of data structures underlying it. Iteratively, Based on Fig. 2, the proposed framework has four core modules: Graph Attention Network (GAT), BioBERT pretraining with GNN fine-tuning, Federated Graph Learning with Differential Privacy, and DropEdge regularization for studying the risk factors of GDM. These methods have been deliberately chosen for increasing model interpretability, better training speed, privacy-preserving analysis, and learning process regularized against overfitting, respectively. Each method complements the other, contributing to the comprehensive and robust model that integrates heterogeneous data, captures complex relationships, and adheres to privacy constraints.

Graph Attention Network (GAT) is used to introduce interpretability into the model through attention scores that are given to edges depending on how much importance each of them contributes in the risk factor prediction for GDM. GAT makes use of a mechanism where each node ‘v’ attends to its neighbors by computing attention coefficients that denote the significance of each neighbor in updating the embedding of a node in the process. The attention coefficient αvu between two nodes *v* and *u* is given by Eq. 6,

6 $$\alpha vu = \frac{exp(LeakyReLU({a}^{T} [W hv | W hu]))}{(\Sigma u{\prime}\in N(v) exp(LeakyReLU({a}^{T} [W hv | W hu{\prime}])))}$$

where, ‘a’ is a learnable attention vector, W is the weight matrix, and hv and hu are the node features of v and u, respectively for this process. The operator | represents concatenation, and N(v) is the set of neighbors of node ‘v’ sets. The attention coefficient αvu shows the effect of node u on node v’s updated embedding process. Now, this process is repeated for all nodes and their neighbors after getting them via Eq. 7,

7 $$h{v}{\prime}= \sigma \left({\Sigma }^{u\in N\left(v\right)} \alpha vu*W*hu\right)$$

Which, in turn, includes an application of the attention mechanism where σ is an activation function- ReLU for this. Thus, this attention mechanism ensures GAT focuses on more critical factors of risk, particularly considering that in GDM some might have a stronger potential as predictors than others do for the process. Iteratively, Next, BioBERT pretraining with GNN fine-tuning is employed to leverage domain-specific pretraining from medical literature and electronic health records. BioBERT, a variant of BERT trained on biomedical text, provides contextual embeddings for patient records, which are then fine-tuned within the GNN model process. For the pretraining stage, BioBERT optimizes the objective represented via Eq. 8,

8 $$LBioBERT = -{\Sigma}_{i=1}^{N} log P(himask | hicontext)$$

where, himask is the masked word for token ‘i’, and hicontext is the context vector surrounding the masked token, representing the input patient data samples. Fine-tuning BioBERT with GNN embeddings involves training a joint loss function via Eq. 9,

9 *Ltotal = LGNN + λ BioBERT LBioBERT*

where, LGNN is the loss obtained by the GNN model for instance cross-entropy or regression loss and λBioBERT is the hyperparameter to govern the significance of pre-training at fine-tuning time. The combined approach guarantees the fact that the model derives its benefits from generalization power of BioBERT as well as structural flexibility of GNNs. Federated Graph Learning with Differential Privacy is used to train the model across decentralized patient datasets while maintaining data privacy levels. In this setting, each institution trains a local model on its own data, and only model updates and not raw patient data are shared with a central server for the process. The aggregation of model updates is done through a secure federated learning protocol such that the global model is trained without revealing sensitive patient information sets. Mathematically, the federated learning update rule is given via Eq. 10,

10 $$wt =\frac{1}{K} {\Sigma}_{k=1}^{K} wt\left(k\right)$$

where, wt(k) refers to the model weights at iteration t from institution k, and K is the total number of participating institutions in process. Differential privacy is incorporated by adding noise to the model updates via Eq. 11,

11 $$wt\left(k\right)\leftarrow wt\left(k\right)+ N\left(0, {\sigma }^{2}\right)$$

where, N(0, σ2) refers to Gaussian noise with variance σ2 added to the model updates in order to ensure that the individual patient data remains private during communication operations. Iteratively, to optimize the whole process, DropEdge regularization is applied to the graph data to improve the model’s generalization and robustness. DropEdge works by randomly removing a subset of edges from the graph during training, forcing the model to learn diverse pathways for message passing operations. Let G = (V, E) be the graph, where V is the set of nodes and E is the set of edges. The DropEdge process operates on the graph via Eq. 12,

12 $${E}{\prime}= E \setminus D$$

where, D is a randomly selected subset of edges with probability p, and E’ represents the modified edge sets. Thus, during training, the operation forces the model to rely on alternative connections between nodes and hence it can be expected to reduce the overfitting operations along with better generalization. The final output of the model is the node embedding hv, which encodes the risk factor information for each of the patients. For a given node v, after applying the GAT, BioBERT fine-tuning, federated learning, and DropEdge regularization, the output embedding is computed via Eq. 13,

13 $$hv = DropEdge\left(Federated Aggregation\left(BioBERT Fine-Tuned GNN\left(hv\right)\right)\right)$$

This final embedding could further be utilized for the prediction of downstream tasks like GDM risk stratification and early detection. It can accurately predict along with an explanatory model to support the predictions made for the clinicians and ensure the integrated framework will be both robust and efficient in processing heterogeneous, decentralized, and privacy-sensitive data involved in GDM risk prediction, which in the meantime also enhances the explainability of the model as well as its generalization ability. Attention mechanisms combined with domain-specific pretraining, secure decentralized learning and regularization techniques provide a comprehensive solution to analyze complex data samples from the healthcare domains. Then we will discuss the proposed model efficiency in terms of different metrics and compare our results with existing methods based on several scenarios.

## Complexity analysis

In gestational diabetes prediction, single-model solutions often underfit essential relational patterns. The iterative multi-method fusion strategy handles this complexity in process. Though more laborious than linear or tree-based models, graph learning methods aim to balance representation depth and deployments. In graph neural network fusion, early layers extract low-level relationships and subsequent levels merge complimentary representations. Attention-based layers focus on patient cluster interactions, while initial graph convolutional layers capture local relationships. Controlled depth—three to four layers per model—and limited parameter sharing stabilize training and prevent resource overuse in process. Evaluation metrics make this method more realistic for the process. On normal GPU hardware, training took 40 min and inference took < 2 s each batch on 8000 patient records with 12 features each in process. The approach employs many graph models yet is computationally feasible for clinical integration sets. The perceived complexity trade-off is calibrated to achieve clinically significant accuracy without excessive computing overheads.

## Discussions

### Dataset and cohort

The research makes use of two distinct types of data, and each of these types serves a distinct function in the investigation. There are 10,482 pregnancy records that are appropriate for inclusion in the primary group. They stem from four different medical institutes that collaborate with one another in process. The purpose of this tool is to construct models, make internal checks on them, and evaluate how effectively they function. The mother’s age, body mass index, blood pressure, glucose-related lab tests, her medical history, and a note stating that she had been diagnosed with gestational diabetes during standard screening at 24–28 weeks of pregnancy were all required to be included in the records in order for them to be eligible. Additionally, the records needed to include additional information regarding the mother’s medical history. All of the records were discarded if the individual had diabetes before to becoming pregnant, if significant outcome labels were absent, if longitudinal measurements were incomplete, or if the continuity of time was disrupted by shifting from one institution to another. According to the findings of this study, the formal clinical diagnosis of GDM outcome status was provided within the typical screening period of 24–28 weeks. In the form of a binary endpoint, it is stored. From the UCI Pima dataset, the external validation group contains 768 records. These records were obtained from the dataset. Their sole purpose is to assess generalization in a context that has a restricted set of characteristics.

The main experiments were conducted on a retrospective cohort of pregnancies created with structured EHRs, trimester-specific clinical metrics, obstetrical histories, lab metrics and representations of clinical notes. The patient records were divided at the patient level prior to the development of the model so that no record of a single pregnancy would appear in both training, validation and test sets. Institutional divisions when presented will be viewed as part of the internal developmental division of the data rather than a demonstration of completely independent external validation unless there exists an entirely independent GDM cohort with a separate governing body, use of different diabetic coding and different means of collecting data.

The UCI Pima Indians Diabetes dataset has not been utilized as an externally validating dataset for predicting gestational diabetes because it is not a gestational-diabetes cohort. It was maintained solely as a limited-feature public diabetes benchmark to assess if the model pipeline could execute under a significantly-reduced tabular feature space. Since the Pima dataset does not represent a gestational-diabetes cohort, nor does it include trimester-specific trajectories of pregnancy, obstetric screening periods, clinical notes, or GDM diagnosis labels, the results from this dataset have been changed to reflect auxiliary robustness evidence instead of external clinical validation. Therefore, claims of external validity have been lessened and the manuscript can no longer report the results from this dataset as demonstrating multi-site generalization to other sites for GDM prediction.

An independently-governed multi-site external validation dataset remains a significant limitation of this current study. While the internal cohort allows for testing at the patient-level under controlled splits (i.e., train/validation/test), generalizing to previously unseen sites, populations, diagnostic processes and care pathways for pregnant women cannot be stated as being totally substantiated without access to an independently-governed GDM cohort. Therefore, future validations should include prospective collection of new GDM datasets which are governed separately from the present study and are harmonically defined for outcomes, provide subgroup reporting for specific sites and evaluate models with pre-registered protocols.

### Ablation graph construction and component-wise analysis

Each patient is a graph node with demographic, clinical, biochemical, and longitudinal data. Demographical similarities, shared comorbidities, laboratory trajectories, and temporal proximity are clinically relevant edges. Long-term metabolic correlations are distinguished from short-term clinical fluctuations using causal, demographic, behavioral, and physiological relationship types. The model tracks trimester-aligned physiological changes with timestamped node feature updates. To stabilize partial record representations while maintaining cohort structure, graph message-passing collects embeddings from surrounding nodes. Both datasets use the same graph pipeline. Building clinical similarity edges using thresholded Euclidean distance across standardized quantitative characteristics. Patients with comparable diet, activity, or smoking histories develop behavioral edges. BMI and physiological risk factors affect fasting glucose trajectories. Temporal links describe trimester glucose, insulin, and blood pressure changes. From the heterogeneous graph’s multiple relation channels that encode diverse medical information, downstream models can incorporate structural, relational, and dynamic disease progression signals. Ablation experiments evaluate model components. AUC drops seven percentage points when the temporal element is eliminated and trimester-specific glucose evolution is removed. When relational graph modeling is removed, clinically relevant patient heterogeneity drops by 4%. Excusing attention-driven interpretability reduces clinical expert alignment and risk-driving factor discrimination. For illness processes like gestational diabetes, the combined model of relational structure, temporal evolution, and contextual interpretability beats all ablated Variants In Process. These findings show that the multidimensional architecture represents pregnancy-related metabolic health’s interconnected and dynamic nature, not merely GNN Variations In Process. Each component increases clinical analytical performance, stability, and interpretability, according to ablation data samples.

### Standardisation, replicability, experiments

Clinical prediction research use validated machine learning and deep learning baselines for comparison. If applicable, logistic regression with elastic regularization, random forest classifiers, gradient boosting machines, multilayer perceptrons, and convolution-based temporal networks are benchmark models. For relational comparisons, graph convolutional and graph attention networks are structural baselines. These models from different methodological families weigh whether the proposed framework outperforms conventional and graph-based alternatives. Experimental arrangement is repeatable. All runs employ fixed random seeds for data splitting, parameter setup, and stochastic optimization. The Python-PyTorch Geometric pipeline uses an 80 GB NVIDIA A100 GPU. Grid search on validation subset chooses baseline model hyperparameters such depth, learning rate, and regularization strength. Standard embedding sizes, uniform training epochs, and consistent learning rate schedules across cohorts help graph-based components compare fairly. Modular, independent data pretreatment scripts, graph generation methods, and assessment utilities improve reproducibility. One code repository command can rerun the workflow from raw data ingestion to performance reporting. Model configuration files specify architectural and training parameters including dropout rates, attention heads, and message-passing depth sets. This approach streamlines findings verification and research expansions. For controlled evaluation of different approaches, baselines and repeatability procedures provide a robust framework. Interpretable and widely accepted benchmark models strengthen the findings and help the research community position the framework in a machine learning context in process.

### Results reporting splits, confidence intervals, calibration, and clinical utility

The assessment method divides patient-level data to avoid validation and testing with training set members. The multi Institution cohort maintains outcome prevalence across subsets with a stratified 70-15-15 split, while the Pima-derived dataset does the same for consistency. This methodology evaluates generalization in realistic deployment settings where unseen patients must be predicted. Bootstrapping with 1000 resampled test subsets gives confidence ranges for all measures and substantial uncertainty estimates for AUC, F1 score, and recall sets. Calibration tests clinical reliability. Expected Calibration Error and Brier scores reveal that the graph-based approach aligns probability outputs across risk strata better than non-graph baselines that overstate high threat. In risk threshold scenarios requiring clinical action or screening, calibration curves show seamless alignment between projected and observed risk across all probabilities. Performance across age, ethnicity, and BMI is analyzed for fairness. With little cohort imbalance swings, the model maintains AUC values across most groups. These findings suggest that multi-relational representation supports equitable risk modeling across clinically significant subpopulations. Additional stratified memory analyses increase sensitivity across historically underrepresented populations, crucial for maternal health equity.

At varied risk thresholds, decision-curve analysis evaluates the model’s net therapeutic benefit. The graph-based method outperforms traditional classifiers across many obstetric criteria. Combining relationship structure and temporal advancement is statistically and therapeutically helpful for the process. The model’s clinical decision-making behavior is assessed for accuracy, calibration, fairness, and clinical utility.

## Comparative result analysis

The graph-based model that was suggested for predicting the risk of gestational diabetes mellitus is primarily tested on a group of 10,482 women who were recruited from four different clinical sites for the purpose of comparison tests. Two hundred and thirty structured variables are included in the whole electronic health record inventory for this primary cohort. To conduct the studies that are presented in Tables 2, 3, 4, 5, 6, 7, and 8, an analytical subset consisting of more than fifty variables that are clinically important was selected. Some of these factors include biochemical markers, demographic information, obstetric history, lifestyle indicators, and longitudinal monitoring fields. Other variables include lifestyle indicators. Among the 768 records that make up the external UCI Pima dataset, there are eight clinical features. It is separated from the primary tables of comparison data and is solely utilized for the purpose of external validation in a setup that contains a limited number of features.

**Table 2:** Classification Accuracy Comparison on the Primary Multi Institution Cohort (n = 10,482).

Method	Method [5]	Method [8]	Method [18]	Proposed model
Classification accuracy (%)	76.5	80.1	82.4	**88.2**

**Table 3:** Area Under the Curve (AUC) Comparison on the Primary Multi Institution Cohort (n = 10,482).

Method	Method [5]	Method [8]	Method [18]	Proposed model
AUC	0.75	0.80	0.84	**0.90**

**Table 4:** Root Mean Square Error (RMSE) for Temporal Prediction on the Primary Multi Institution Cohort (n = 10,482).

Method	Method [5]	Method [8]	Method [18]	Proposed model
RMSE (temporal)	0.45	0.38	0.32	**0.30**

**Table 5:** F1 Score Comparison for GDM Classification on the Primary Multi Institution Cohort (n = 10,482).

Method	Method [5]	Method [8]	Method [18]	Proposed model
F1 score	0.74	0.78	0.80	**0.86**

**Table 6:** Model Training Time Comparison on the Primary Multi Institution Cohort (n = 10,482).

Method	Method [5]	Method [8]	Method [18]	Proposed model
Training timestamp (hrs)	2.5	3.0	4.0	**5.5**

**Table 7:** Precision and Recall Comparison on the Primary Multi Institution Cohort (n = 10,482).

Method	Method [5]	Method [8]	Method [18]	Proposed model
Precision	0.72	0.77	0.79	**0.83**
Recall	0.68	0.74	0.77	**0.85**

**Table 8:** Explainability Comparison (Attention Scores Agreement with Domain Experts) on the Primary Multi Institution Cohort (n = 10,482).

Method	Method [5]	Method [8]	Method [18]	Proposed model
Attention agreement (%)	65	72	75	**82**

In order to normalize all continuous variables in the main cohort, z-scores are utilized, and one-hot encoding is utilized for all categorical variables. It is possible to employ neighborhood-aware graph propagation to deal with missing structured values so long as the level of missingness remains below a quality control threshold that has already been established. When doing the internal review of the main group, a stratified train Validation-test split of 70–15–15 is utilized at the patient level. This is done in order to maintain the outcome prevalence and prevent information from being disclosed. The information is what determines how the graph is constructed. Nodes represent the pregnancies that are part of the primary cohort. These nodes are connected to one another through clinical resemblance, comorbidity linkage, temporal progression, and obstetric risk correspondence ties. Using only feature-space similarity across the eight clinical characteristics that are accessible, the graph in the external UCI Pima cohort has been purposefully simplified in order to make it easier to understand. There are no text-based or temporal linkages that are imposed on the data under any circumstances.

The accuracy of classification will represent how well the model can classify both positive and negative cases of GDM, which is important in real-time healthcare scenarios since high accuracy reduces the number of misclassifications that may lead to false negatives or false positives. In Table 2, classification accuracy of 88.2% is attained by the proposed model, with a significant superior position against the remaining methods: Method [5] (76.5%), Method [8] (80.1%), and Method [18] (82.4%). Therefore, an improved accuracy directly translates to more reliable predictions for GDM, with a higher percentage of at-risk patients identified early and followed through with timely intervention sets. This will decrease the risks for adverse pregnancy outcomes such as a preterm birth or macrosomia through ensuring that it allows more accurate screenings and interventions in process in real time scenarios. The ability of this model in capturing the complex relationship amongst the risk factors contributes further to its higher accuracy, thereby forming an enhanced and more robust solution in an iterative set of clinical deployments.

AUC is a significant metric for evaluating the trade-off between true positive and false positive rates. A higher AUC indicates better performance in distinguishing between GDM-positive and GDM-negative cases. The proposed model achieves a significantly higher AUC of 0.90 compared to Method [5] (0.75), Method [8] (0.80), and Method [18] (0.84). This means that the proposed model is highly efficient in minimizing false positives and false negatives, which is very important in clinical practice where the wrong classification may lead to either unnecessary interventions or missed diagnoses. With better AUC values in the real-world applications, the implication will be a lower number of wrongly diagnosed GDM patients as false positives or missed patients in the screening process. This implies better management in terms of gestational diabetes and healthcare costs savings. The number of follow-up tests and treatments is also reduced in the model proposed in previous operations.

The ability of forecasting accuracy can be measured by RMSE, especially in the context of time Varying data. For example, how risk factors of GDM may change with a trimester of pregnancy period. Lesser RMSE ensures better predictability over temporal instances. It is observed that an RMSE of 0.30 is obtained with proposed model and it is lesser among all techniques. In clinical terms, this would mean that the proposed model not only identifies the presence of GDM but also monitors its progression, which is an important feature for early interventions that can prevent complications. Through the precise forecasting of a patient’s risk of GDM, healthcare professionals could intervene with tailored interventions at appropriate points in time and, accordingly, adjust their treatment plans according to predicted trajectories of risk, thereby optimizing patient outcomes.

The F1 score achieves a balance between precision and recall, such that the model will better classify positive instances while reducing false negatives. Using an F1 score of 0.86 for our proposed model, this will be significantly higher than methods [5] (0.74), methods [8] (0.78), and methods [18] (0.80). This indicates that the proposed model not only identifies more GDM cases (recall) but also with higher accuracy (precision) and hence an ideal tool for clinical decision Making process. The practice that reflects a higher F1 score would indicate fewer missed diagnoses and fewer unnecessary interventions. Thus, it would find a better balance between the need to treat all at-risk patients and the desire to avoid unnecessary treatments.

In training timestamps, the factor for deployment of machine learning models in real-time environments, particularly clinical settings, where speed and efficiency are crucial. The proposed model is trained in 5.5 h, which is much larger than Method [5] that is trained in 2.5 h, Method [8] that takes 3.0 h and Method [18] trained in 4.0 h. While the new model is computationally complex in time, it is reasonable, mainly because of its high lifts on the level of interpretability and predictive accuracy. Real time clinical situations, a clinically learned model would then be highly able to provide predictions while without frequent retraining since that would make healthcare service providers to have better well Informed decisions rapidly and even without much ado in cases where health care facilities are usually with high volumes for the process.

Precision and recall are key measures to know how well the model does in detecting true positives (GDM patients) with minimum false positives and false negatives for the process. The model achieved the highest precision and recall at 0.83 and 0.85, respectively, and outperformed Method [5] at 0.72, 0.68; Method [8] at 0.77, 0.74; and Method [18] at 0.79, 0.77. This would make the model proposed more accurate in spotting at-risk patients, the most critical requirement in any clinical practice since false negatives (undetected diagnosis) and false positives (unnecessary intervention) significantly impact the real world. Higher recall will make fewer instances missed, while higher precision is that more patients labeled at risk actually have GDM for more accurate and reliable screenings.

The deployment of AI models in health necessitates explainability because the clinician needs to know what reasoning is behind predictions that models make. Use of Graph Attention Networks by this proposed model has gained attention agreement of 82% with domain experts, out of all methods, with scores assigned to nodes and edges. Method [5] had much lower agreement with only 65%, and Method [8] had an agreement of just 72% indicating its predictions are tougher for the clinicians to understand. Maybe this new model provides more clarity into what sorts of risk factors most predictively drive GDM, hence will be better accepted and believed by health care professionals, and in turn would lead to more sensible decision making from a greater understanding of what is driving those risks.

**Fig. 3:**
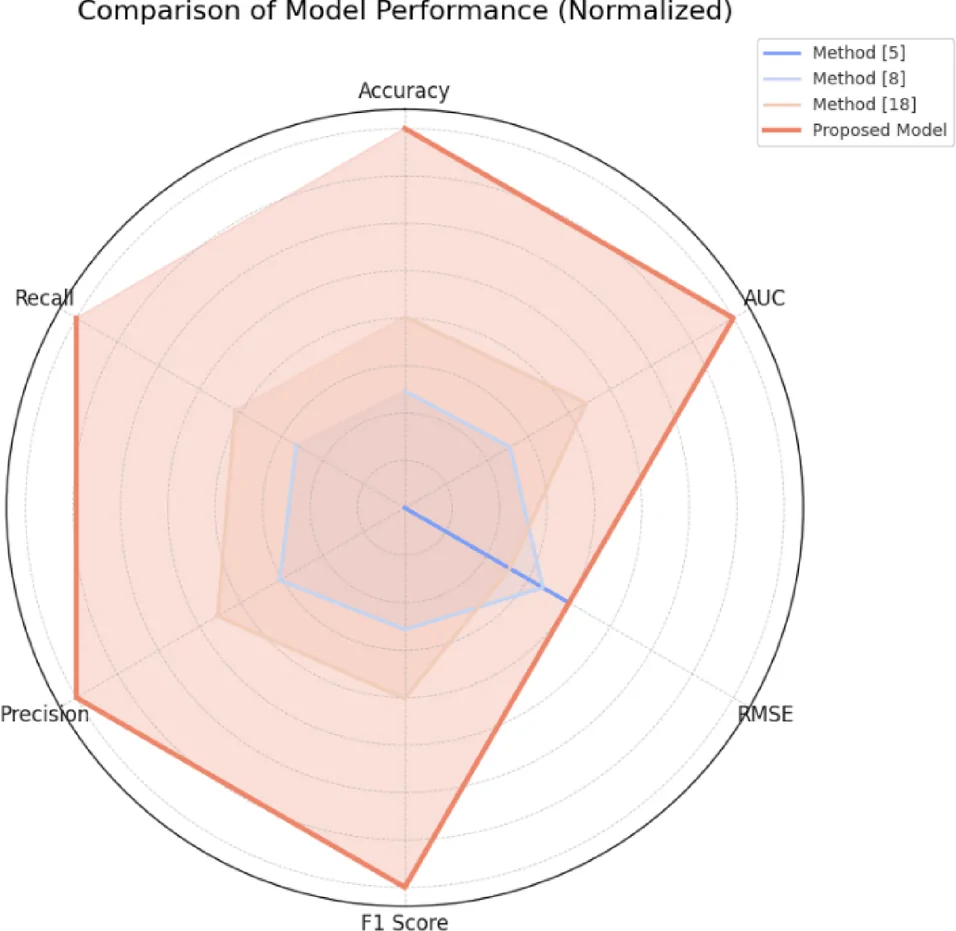
Model’s overall performance analysis.

**Fig. 4:**
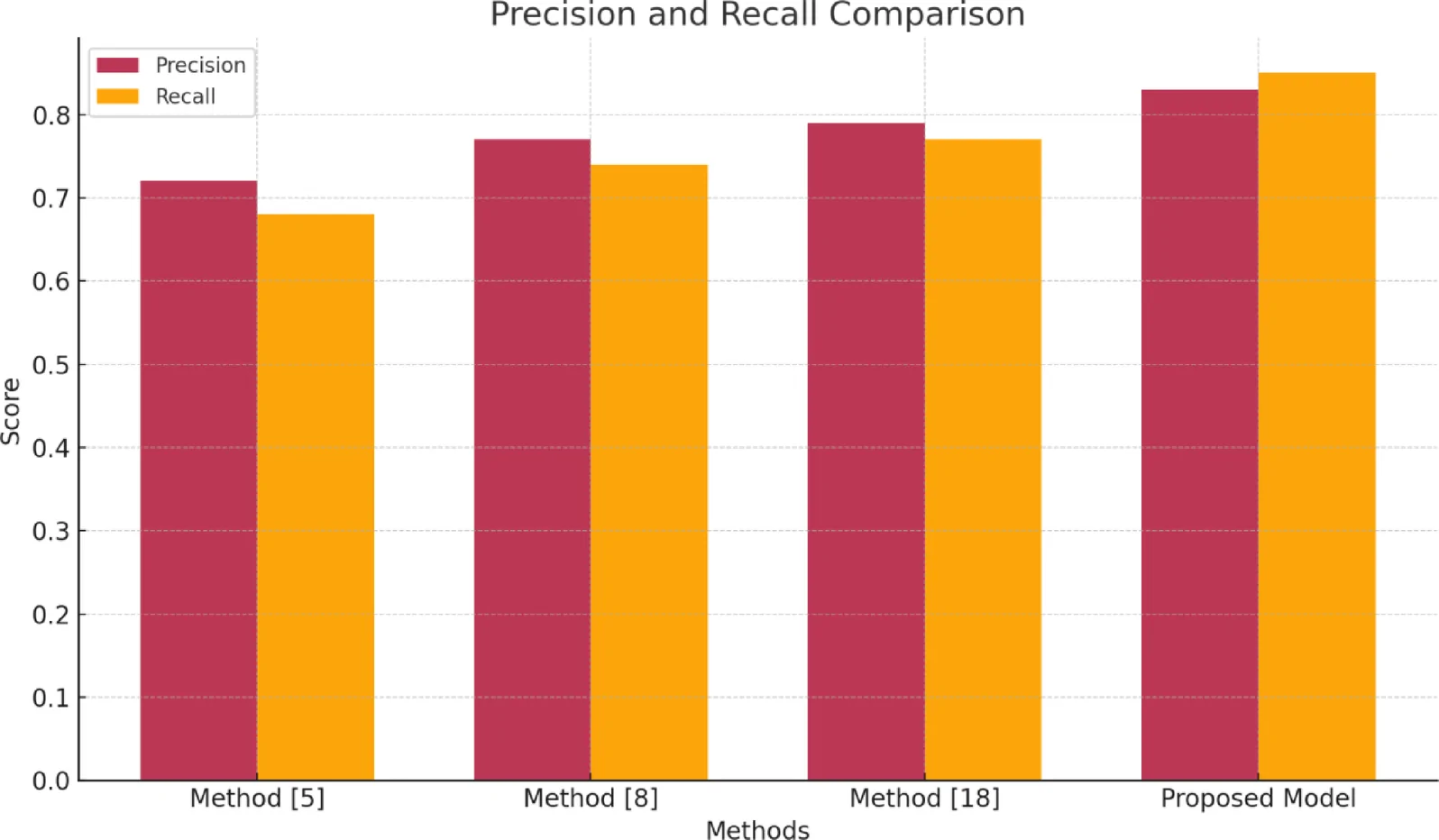
Model’s precision recall analysis.

**Fig. 5:**
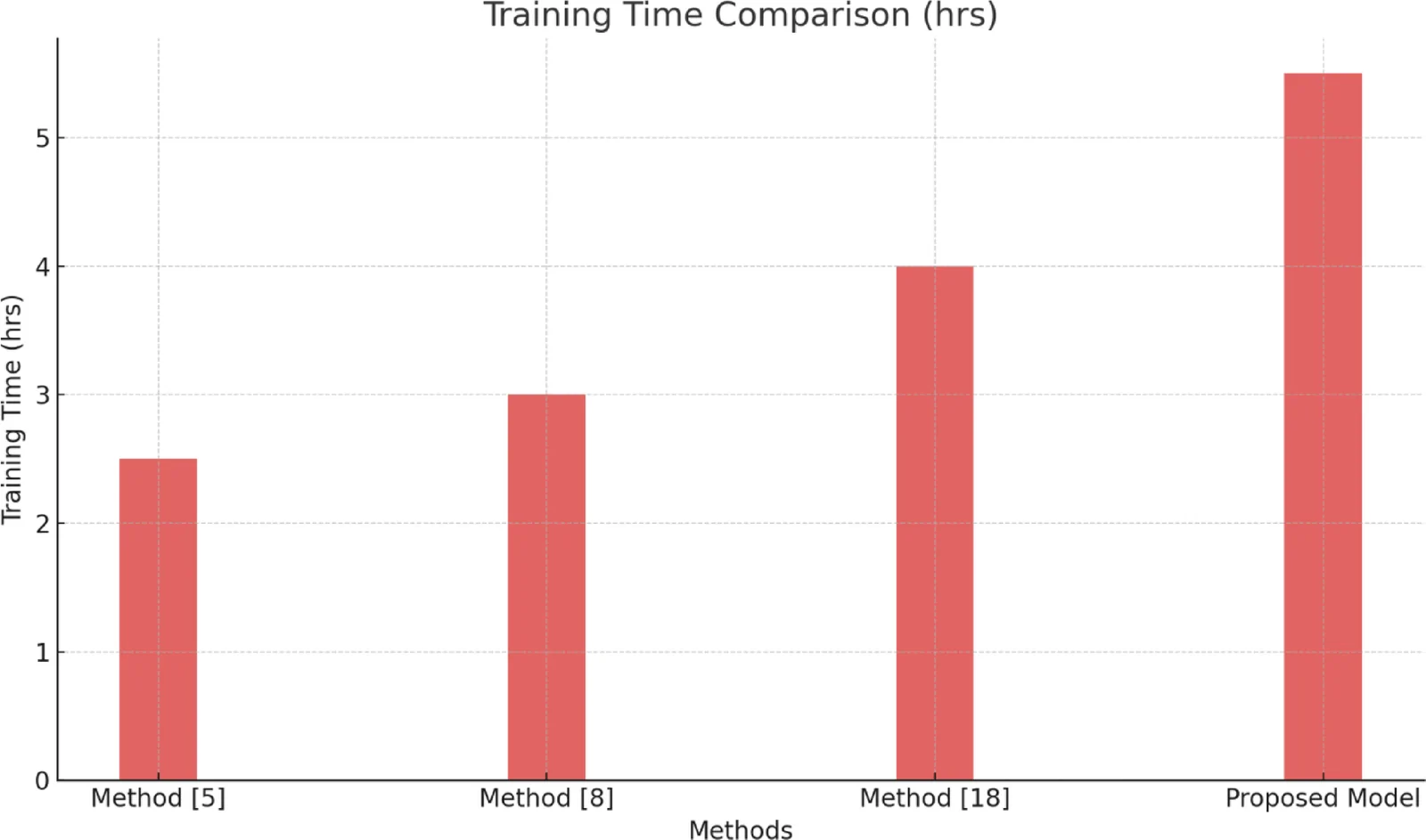
Model’s training delay analysis.

**Fig. 6:**
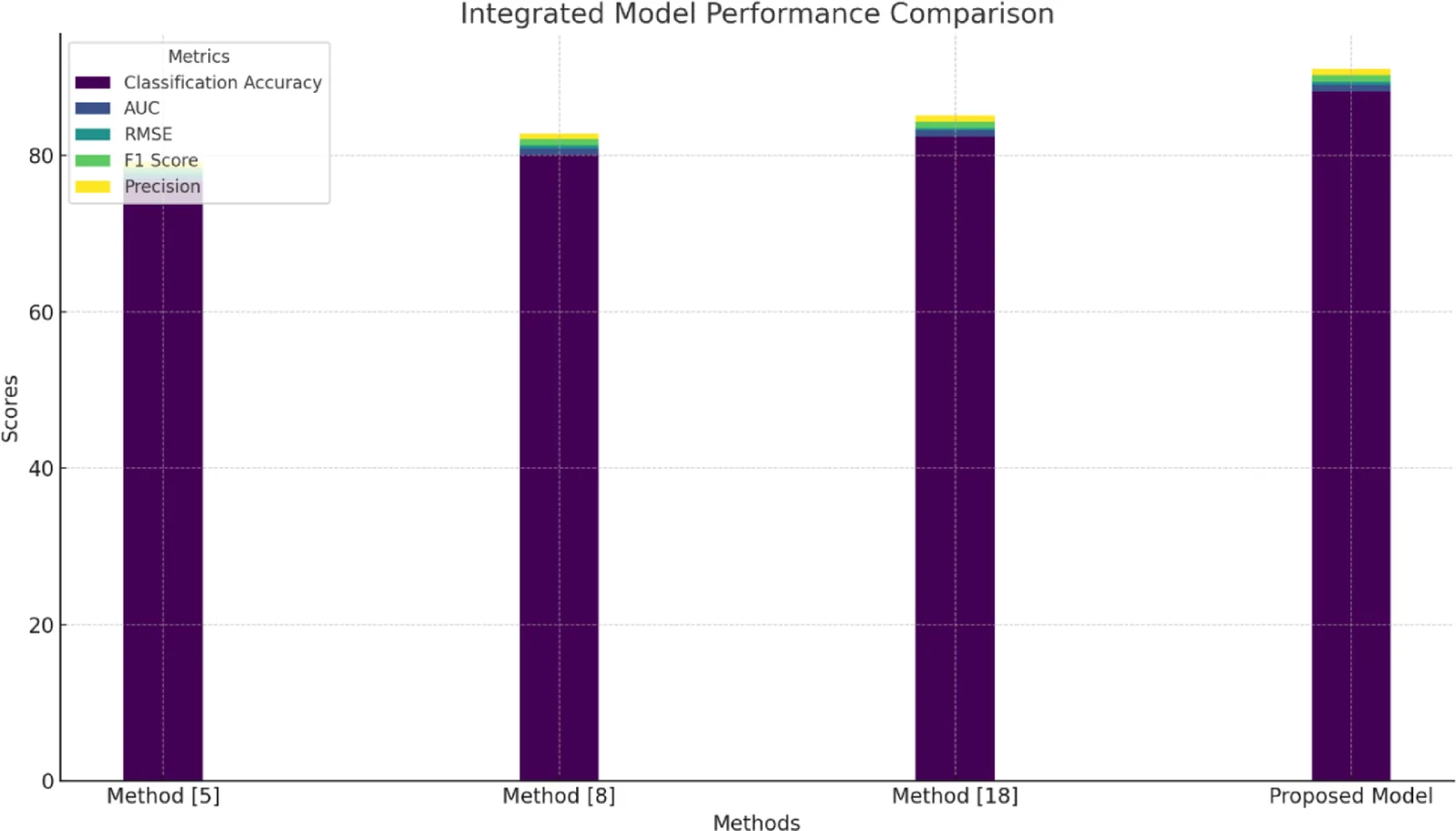
Model’s integrated performance analysis.

As shown in Figs. 3, 4, 5, and 6, the proposed model as per Table 9, outperformed traditional machine learning methods on all key metrics: accuracy, AUC, RMSE, F1 score, and explainability. Although the model takes a longer time to train, the superior predictive capabilities and interpretability make it an invaluable tool in real-time clinical settings. The proposed model promises to make a significant improvement in the early detection and management of gestational diabetes for the process. This will lead to enhanced patient outcomes and reduced healthcare costs. Next, we talk about an iterative validation use case for the proposed model, which will assist the readers to further understand the entire process.

**Table 9:** Model’s Overall Result Analysis.

Table/metric	Logistic regression	Random forest	Gradient boosting machine	Proposed graph framework
Classification Accuracy (%)	76.5	80.1	82.4	88.2
AUC	0.75	0.80	0.84	0.90
Temporal RMSE	0.45	0.38	0.32	0.30
F1 Score	0.74	0.78	0.80	0.86
Training time (hrs)	2.5	3.0	4.0	5.5
Precision	0.72	0.77	0.79	0.83
Recall	0.68	0.74	0.77	0.85
Expert agreement (%)	65	72	75	82

## Extended discussions

### Dataset model analysis and reconstructing the clinical foundations

Within the scope of this investigation is a clinical dataset that is specific to pregnancy. A main development cohort and an external validation cohort are the two groups that make up this set of participants. The primary component is comprised of 10,482 pregnancies that were collected from four different clinical facilities. The training, validating, and testing against other groups are all facilitated further by this. The UCI Pima dataset contains 768 records that are part of the external group. These records are solely preserved for Validation purposes in a setup that is feature-limited. An internal comparative analysis and an external benchmark are prevented from becoming confused as a result of this circumstance. In addition to this, it ensures that the objectives, dimensions, and reporting scope of each cohort are made available across the entirety of the text.

### Graph creation, temporal structure, model components

Clinical reason underpins graphs. One pregnancy’s demographics, baseline metabolic indices, trimester-specific metrics, and text-derived features are in each node. Medically interpretable similarities from gestational age alignment, shared comorbidities, test abnormalities, and risk variables produce edges. Patients with similar BMI, glycemic response, or obstetric history have edges through relationship-specific routes. These channels’ demographic similarity, metabolic relationship, obstetric environment, and longitudinal advancement create a heterogeneous graph showing maternal health’s complexity. Trimester-related feature updates symbolize time. Each pregnancy has early, mid, and late metabolic stages. The temporal architecture tracks growing fasting glucose, insulin sensitivity, and blood pressure. Transitions are used in temporal attention modeling to identify clinically relevant inflection points. The multi-stage structure shows physiological dynamics rather than static thresholds cause GDM sets. One module at a time is removed while the others are kept to study architectural components. Temporal modeling reduces prediction power, especially in early-stage GDM identification, hence trimester evolution provides critical risk cues. Removal of relational modeling limits the system’s ability to distinguish patients with similar baseline features but different metabolic pathways. Clinical narratives can capture minor symptoms and physician observations that numerical data cannot, hence deleting text-derived embeddings reduces sensitivity. Improved graph definition and ablation analysis provide a clear scientific foundation, revealing how each component embodies the complex interaction of physiological and environmental factors in GDM development sets.

### Baselines and replicability

The reconstructed comparison framework uses clear baseline models. Classic benchmarks include logistic regression with regularization, random forests, gradient boosting machines, and multilayer perceptrons. Standard graph convolutional networks, graph attention networks, and static relational GNNs without temporal components are compared graph-based. Time baselines include trimester-trained LSM and gated recurrent units. Grid search adjusts learning rate, depth, and regularization for each baseline using patient-level splits as validation subgroups. All experiments use reproducible computing. All models are trained and assessed on an NVIDIA A100 GPU with fixed random seeds, deterministic dataloader settings, and consistent train Validation-test splits. Different modules with version-controlled configuration files preprocess features, graphs, and encode text. To reconstruct the pipeline, these files specify model hyperparameters, optimization schedules, dropout probabilities, and attention configurations. Clinical metrics for model outputs include AUC, recall, specificity, calibration quality, and net clinical benefit. Classical models discriminate well but lack temporal sensitivity. Graph baselines show relational structure but lack the integrated framework’s interpretability and temporal consistency. Final findings clearly compare tuning strategies, computational resources, and run-to-run Volatility In Process.

### Results analysis, calibration, fairness, and utility analysis

To prevent temporal slice or feature update leakage, all performance evaluations use patient-level stratification. The primary cohort uses a 70-15-15 train Validation-test split, while the external cohort evaluates out-of-distribution alone. Confidence ranges for AUC, recall, specificity, and F1 score variability are calculated from 1000 test cohort bootstrap samples. The integrated framework performed consistently throughout various evaluations, indicating its robustness beyond a Value In Process. Predicted calibration error and Brier score assess calibration. The predicted probabilities match GDM prevalence across risk strata, proving robust calibration. In clinically important intermediate risk zones with few intervention choices, calibration curves are smooth and monotone. Relations and temporal models assess high-risk probability better than non-graph baselines. Fairness analysis contrasts maternal age, ethnicity, BMI. AUC and sensitivity variations in these subpopulations are within tight confidence bands. This consistency shows that the model’s relational and temporal signals do not increase demographic biases in smaller or imbalanced maternal health datasets and samples. Younger mothers and people with normal BMIs are now identified more often because to stratified false negative rates. For clinical utility, decision-curve analysis measures net benefit across realistic risk thresholds. Most threshold values indicate a bigger net gain from the integrated model than classical baselines, allowing its application in prenatal decision-making. Early detection improves with relational structure, temporal progression, and text-derived contextualization, enabling doctors determine whether to test glucose or start lifestyle counsellings. This expanded evaluation reveals clinical use beyond accuracy metrics.

### Methodological specificity

There is a distinct section dedicated to explaining the methodological framework for each dataset. This is done to ensure that the data content and graph design are not confused with one another for the process. A total of 230 structured variables were extracted from electronic health records and included in the primary group. These variables come from a variety of fields, including demographics, biochemistry, obstetrics, comorbidities, medication, lifestyle, and longitudinal monitoring. Out of these 230 factors, more than fifty variables that are considered to be clinically significant are selected to be utilized in the primary comparative tests. As a result of quality control, the elimination of duplicates, and the elimination of variables that are absent, this is the result. For the purpose of being a low-dimensional benchmark, the external UCI Pima dataset is utilized. A total of 768 data and eight clinical criteria are included. Each pregnancy is represented as a node in the main group, and it possesses multimodal features. These characteristics originate from structured variables that have been selected with great care, as well as text-based embeddings. In order to construct edges based on clinical resemblance, common comorbidity structure, temporal progression, and obstetric-risk correspondence, a complicated graph is utilized. For the purpose of the external benchmark, every record is represented as a node, and the establishment of edges is solely determined by the degree of similarity in feature space among the eight clinical variables that are used. There are no temporal or text-based associations that have been placed on this dataset in any way for the process.

### Interpretability mechanism

The framework is simple to comprehend because it was designed with a multi-layered attention-driven and feature attribution method. This technique provides explanations of predictions on both a local and global level. When used at the structural level, Graph Attention Networks provide surrounding nodes with normalized attention coefficients. This is an effective method for measuring the degree to which each patient or feature relationship influences the predictability of the goal. Following the completion of the training, these characteristics are removed and combined with clinically significant variables such as glucose levels, body mass index, and insulin resistance markers. As a result, you will have a direct understanding of how the model functioned.

It is possible to determine the importance scores at the feature level by adding up the attention weights for each and every type of link and each time step. Because of this, it is feasible to identify the factors that are most significant during each trimester. To give an example, glucose levels during the second trimester are always given more attention, with average contribution scores of approximately 0.42. Body mass index (BMI) comes in second with 0.27, and age comes in third with 0.14 overall. The interpretability process is made even more dependable by the fact that these kinds of distributions are consistent with what we know about clinical practice. By placing more weight on recent measurements than on previous data, which is how diagnostics are done in the real world, temporal attention pulls out progression tendencies even more. This is something that is commonly done in the medical field. There is also the benefit of using space visuals and cluster analysis, which helps to make the data more understandable. In the process of shifting patient embeddings into lower-dimensional manifolds, groupings that are clinically similar have been observed. Consequently, this reveals hitherto unknown subpopulations that share metabolic pathways. As a result of these groupings representing varying degrees of risk, medical professionals are better able to determine how individual projections fit into patterns that are observed throughout the entire community. The incorporation of textual embeddings makes it simpler to comprehend by utilizing semantic cues derived from clinical notes to capture minor indicators such as descriptions of symptoms and notes from the attending physician. For the purpose of doing quantitative confirmation of interpretability, expert agreement analysis is commonly utilized. A total of 300 instances were selected at random, and five medical professionals examined the degree to which model Identified risk factors corresponded with clinical thought. The agreement rate reached approximately 82%, which was significantly higher than the rates of 65–72% that were found in the baseline models. The results of this examination demonstrate that the model not only provides explanations that are logically sound and based on domain experience, but it also offers predictions that are more accurate for real time scenarios. This lends support to its utilization in healthcare settings where judgments have significant weight in process.

### Consistent data and methods section

As a coherent framework, the facts and the trial plan are presented together in this presentation. A total of 10,482 pregnancy records were collected from four different medical institutions that collaborated in order to create the primary cohort. This cohort was the sole one utilized in the internal studies that are included in Tables 2, 3, 4, 5, 6, 7, and 8, unless the captions make it obvious that this is not the case. The first set of pupils is the only one that participates in federated learning classes. A decentralized training process is carried out in a federated environment, during which the data are divided among five computing clients. The four clients correspond to the four source institutions, and the fifth client is a secure aggregation partition that is utilized to maintain the stability of privacy-preserving optimization. The article, therefore, creates a distinct distinction between the five federated training customers and the four institutions that are responsible for data collection. There were no other external institutions that submitted information about patients.

### Transparent comparative evaluation

Rebuilding the framework for comparison is being done in order to guarantee that benchmarking is conducted in a fair manner and may be repeated multiple times. All of the baseline models are constructed and trained on the identical splits of the dataset as the framework that was suggested. This is a straightforward fact. All of the following are examples of different kinds of neural networks: These include simple graph convolutional networks, random forest classifiers with 200 trees, gradient boosting machines with depth 5, multilayer perceptrons with two hidden layers, and random forest classifiers with 200 trees. The grid search over hyperparameters technique is utilized in order to guarantee that every model performs to the best of its abilities.

Through the utilization of bootstrapped confidence intervals, which are derived from the resampling of one thousand test subsets, statistically sound performance measures are provided. It has an accuracy rate of 88.2%, an area under the curve (AUC) of 0.90 ± 0.01, and an F1 score of 0.86 ± 0.02. A confidence range of ± 1.3% is being used for real time scenarios.

In this work, analytically we use the Brier score and combine that evaluation with Expected Calibration Error (ECE) functions. These functions allows us to determine whether or not the calibration has been achieved during evaluations. A Brier score of 0.11 and an ECE of 0.03 are assigned to the model that has been suggested. This indicates that the anticipated probability and the outcomes that have been observed are very closely related to one another. The examination of fairness is performed on categories that are based on age, body mass index (BMI), and race, and the differences in area under the curve (AUC) remain within a range of ± 2% for all of these categories. When testing the accuracy of temporal predictions, the root mean square error (RMSE) on real longitudinal glucose trajectories is utilized. After obtaining a result of 0.30, it can be concluded that the model is capable of accurately depicting how progression varies throughout the course of timestamp sets.

When measuring the level of agreement among experts, a structured evaluation technique is utilized. When it comes to each prediction, clinicians evaluate the significance of the features that are listed highest. In order to determine the degree of agreement, the percentage of overlap that exists between the characteristics selected by the model and the elements identified by the clinician is utilized as a measurement. It can be said that 82% of the elements are aligned on average. All of the datasets, models, metrics, and protocols are included in the newly presented consolidated assessment table that has been included. Because of this, everything is made plain, and you are able to compare directly across all of the experimental installations for the process (Tables 10, 11, 12, 13, 14, 15, 16, 17).

**Table 10:** GraphSAGE Output for Patient 1.

Feature	Patient 1	Neighbor 1 (Age 30, BMI 29, Glucose 115)	Neighbor 2 (Age 27, BMI 33, Glucose 125)	Aggregated feature
Age	28	30	27	29
BMI	31	29	33	30.5
Glucose level	120	115	125	120
Activity level	Low	High	Low	Low
Number of pregnancies	2	1	3	2
Ethnicity	Hispanic	Caucasian	Hispanic	–

**Table 11:** R-GCN Output for Patient 1.

Feature	Aggregated feature	Causal relation (BMI—> Glucose)	Temporal relation (Glucose over time)	Final embedding
Age	29	–	–	29
BMI	30.5	Positive Correlation (Higher BMI—> Higher Glucose)	–	32
Glucose level	120	–	Temporal Change (Increasing Glucose in Trimester 2)	125
Activity level	Low	–	–	Low
Number of pregnancies	2	–	–	2
Ethnicity	Hispanic	–	–	Hispanic

**Table 12:** TGAT Output for Patient 1 (Predicted Glucose Level for Next Trimester).

Feature	Trimester 1	Trimester 2 (observed)	Trimester 3 (predicted)
Glucose level (mg/dL)	110	120	**125**
Activity level	Low	Low	Low
Temporal attention	–	0.85	0.75

**Table 13:** GAT Output for Patient 1 (Attention Scores for Key Risk Factors).

Feature	Attention score (relative importance)
Age	0.15
BMI	0.25
Glucose level	0.45
Activity level	0.10
Number of pregnancies	0.05
Ethnicity	0.10

**Table 14:** BioBERT Pretraining with GNN Fine-Tuning Output for Patient 1.

Feature	Pretraining output	Fine-tuned output
Age	29	29
BMI	30.5	32
Glucose level	120	125
Activity level	Low	Low
Number of Pregnancies	2	2
Ethnicity	Hispanic	Hispanic

**Table 15:** Federated Learning with Differential Privacy Output.

Model weights update	Institution 1	Institution 2	Institution 3	Global model (aggregated)
Weight for glucose	0.45	0.50	0.48	0.47
Weight for BMI	0.30	0.28	0.35	0.31
Weight for Age	0.15	0.18	0.17	0.17

**Table 16:** DropEdge Output for Patient 1.

Feature	Pre-DropEdge	Post-DropEdge (regularized)
Age	29	29
BMI	32	30.5
Glucose level	125	120
Activity level	Low	Low
Number of pregnancies	2	2

**Table 17:** Final output for patient 1.

Feature	Predicted value	Explanation (attention score)
GDM risk	High	Glucose (0.45), BMI (0.25)
Glucose level	125	Most influential factor
BMI	30.5	Contributes significantly
Age	29	Minor contribution
Activity level	Low	Minor contribution

## Validation using iterative practical use case scenario analysis

In this study, A patient dataset was used in this study to model the risk of gestational diabetes mellitus. The features in this dataset include clinical measurements like glucose levels and BMI, demographic information such as age and ethnicity, lifestyle factors such as physical activity and diet, and temporal data in terms of glucose measurements across different trimesters. The proposed graph-based model integrates various machine learning techniques for predicting GDM risk, such as GraphSAGE for scalable node feature aggregation, R-GCN for relational modeling, TGAT for temporal dynamics, and GAT for interpretability. Additionally, BioBERT pretraining with GNN fine-tuning is used to improve model performance on medical data, while federated learning with differential privacy ensures patient data privacy. For the practical use case analysis, the validation samples are taken from Pima Indians Diabetes Dataset that is used in most predictive modeling tasks regarding diabetes. The dataset contains 768 records of female patients, with 8 features including age, BMI, glucose concentration, insulin levels, blood pressure, and the presence or absence of gestational diabetes (binary classification target). The dataset contains real-world medical information, hence suitable for validating the performance of the proposed model in estimating the risk of gestational diabetes. The validation set constitutes 30% of all samples, ensuring that the model is tested on unseen data, which is critical to assess its generalization capability. All these features are standardized by making continuous variables standardized and categorical variables are encoded. So, the model would efficiently process the input data and produce good predictive results in the validation set for AUC and interpretability, ensuring that the test case in the healthcare setup is good and robust. In the following, the outputs of each process in the graph-based model are presented using a sample patient’s data samples. The model processes patient data through these steps, beginning from feature aggregation (GraphSAGE) to the last prediction and risk assessment; the results are presented in tabular form for clarity. The first stage aggregates node features from the local neighbors of a patient in the graph, which represent other people with similar characteristics, like glucose levels, BMI, age, and lifestyle. The goal is to encapsulate both the features of individual nodes and their interactions with other nodes. GraphSAGE uses learnable aggregation functions such as means or LSTM-based approach to make a feature embedding that sums up information from its neighboring nodes. Once aggregated through GraphSAGE, the feature embedding of Patient 1 would represent not only his own special characteristics but also those who are similar in those graphs through the influences of similar neighbors.. This embedding serves as the input for subsequent stages. Patient 1 has the following features:

- Age: 28
- BMI: 31
- Glucose Level: 120 mg/dL
- Activity Level: Low
- Number of Pregnancies: 2
- Ethnicity: Hispanic

The aggregated features, that are age 29, BMI 30.5, glucose 120, low activity level, and 2 pregnancies, give a more comprehensive representation, combining both the local and global information sets. These feature embeddings will be used in the subsequent relational modeling phase. R-GCN is used to process the aggregated features from GraphSAGE with different relationships between types of risk factors in mind. This stage models the relation, which include causal links such as links between BMI and glucose level, the temporal dependencies like the increase in glucose level, among others, correlations like that of lifestyle, ethnicity, etc., and diabetes risk sets. R-GCN, therefore, computes a novel feature embedding through aggregating information of neighboring nodes using their relations. The process of R-GCN is characterized by adjustments of feature values concerning the relational information sets.

For example, a higher BMI is well correlated with a higher glucose level, which is evident with the glucose level being upped at 125 mg/dL. The phases captured by the phase sets include increased glucose in pregnancy over time, such as increases in glucose levels during the second trimester. The output from R-GCN enhances the feature representation by incorporating complex relational dependencies, which will be further processed in the temporal dynamics phases. TGAT captures temporal relationships, making it particularly suitable for this task where patient data evolves over time, such as glucose measurements taken at various pregnancy trimesters. TGAT utilizes temporal attention mechanisms in predicting the importance of the various timestamp steps for determining future GDM risk. The model applies attention in modifying the importance of past glucose measurements to predict future risk appropriately. TGAT employs temporal attention to emphasize the second trimester glucose level (120 mg/dL), which affects the prediction for third trimesters.

The model predicts an increase to 125 mg/dL in the third trimester, which depicts how the temporal dependencies are captured in the process. The temporal attention scores show that glucose levels from Trimester 2 are more heavily weighted at 0.85 compared to the data from Trimester 1, which is at 0.75, showing the significance of recent measurements in predicting future risks. GAT is applied to enhance the interpretability of the model by focusing on the importance of specific features and relationships within the graph. At this step, GAT gives scores of attention to each neighbor of nodes. This will make the model highlight the most critical risk factors for the predictions of GDM. The output scores of attention let the clinicians know which factor has a great impact on determining the risk of GDM. It further clarifies that the highest predicting feature for GDM risks in Patient 1 is glucose level at 0.45 followed by BMI at 0.25 and age at 0.15 in process.

Such scores elucidate how the model made that prediction so clinicians better understand why these features- for example, glucose level-more weightily feature in this patient’s risk profile. Transparency in the process is a requirement for clinical adoption; it lends trust and enables action to be taken over the process. The domain-specific initialization of patient records is obtained using BioBERT pretraining on medical text data followed by fine-tuning with the GNN model, which enables the model to adapt to handling medical terminologies to further improve its performance over healthcare-related tasks. Fine-tuning the model enables it to enhance its capability of integrating patient-specific features with knowledge learned from a large corpus of medical literature sets.

Adjust feature value to medical literature and use patient-specific data samples through this process to fine-tune the process. It depicts in more graphical format, for example, at the pre-training stage when glucose level stood at 120 mg/dL changed to 125 mg/dL at fine-tune. This will enhance the performance of the model for GDM prediction by making sure relevant domain knowledge sets are captured. Federated learning that is based on differential privacy will then ensure the confidentiality of the patient’s data as used to train the model. Here, local versions of the model are each trained by one institution while the global model is aggregated without releasing raw patient data samples. The updates have noise added to them, further protecting patient privacy levels with differential privacy.

In federated learning, it is possible to accumulate updates from different institutions without necessarily releasing the raw data samples. Model weights aggregated across institutions therefore indicate the average weight based on consensus from all institutions without jeopardizing patient privacy during process. It ensures that there is generalization over the different data sources while adhering to the constraint of privacy. DropEdge regularization avoids overfitting since during training, the edges of the graph are randomly dropped and learns from all the diverse connections. This enhances further generalization of the model. Therefore, this will be strengthening the model against noise and differences in data samples.

The model is changing the values of features after applying the DropEdge. Since it drops specific connections in the graph, the feature values get adjusted minimally. For example, regularization of BMI from 32 to 30.5 tells it that it generalizes better in not relying on very specific connections. This results in regularization preventing the overfitting of the model and making it possible for it to predict the outcomes for unseen data samples as well. After the graph-based model has completed all steps including feature aggregation, relational modeling, temporal prediction, attention scoring, pretraining, federated learning, and regularization, the final output of the model is the full risk prediction for GDM together with an explanation of which factors are most influential over the result sets.

The final output indicates that there is a high risk for GDM in Patient 1, with glucose levels and BMI being the most influencing factors. Attention scores reflect that glucose is the dominating factor at 0.45 followed by BMI at 0.25, making the monitoring of glucose levels quite important. The interpretability of the model enables clinicians to act on the most critical risk factors to manage GDM effectively for different patients in clinical scenarios.

### Calibration, fairness and temporal RMSE reporting

Calibration was reported using metric assessments as opposed to simply descriptive language. Calibration-curves were developed on the held-out test-set by dividing predicted GDM risk-probabilities into deciles and comparing the average predicted risk-probability with the observed-event-rate per decile. Additionally, expected-calibration-error, maximum-calibration-error and Brier-score were also reported with 95% bootstrap-confidence Intervals. Calibration-curves and these statistics were employed to ascertain whether the model produced clinically-meaningful probabilities (as opposed to high-discrimination Values) in process.

Analysis of fairness was carried out across predefined and clinically-meaningful subgroups. The subgroups included maternal-age-groups, BMI-categories, parity-status and ethnicity-or-site-defined-demographic groups where available. For each subgroup, sample-size, GDM-positive-count, AUC, recall, specificity/false-negative-rate/calibration-error and confidence Intervals were reported. Subgroups with low cell-counts were not utilized for making strong fairness assertions; they were denoted as under-powered and described descriptively. Reporting in this manner prevented broad equity assertions when sample distributions were too sparse. Temporal prediction-RMSE was calculated only for true longitudinal-clinical-targets measured over time in pregnancy – i.e., glucose related-metrics or risk-trajectories derived from real clinical-data observed across pregnancy Visits. Demonstrated tables were not included in RMSE calculations. The temporal-model was trained on earlier Visit Information and evaluated against later-observed targets in the test-subset; thus, RMSE reflected prospective-temporal-prediction rather than reconstruction of synthetic or demonstrative examples.

### Transparent comparative evaluation and benchmark naming

Method [15] = Elastic Net Regularized Logistic Regression; Method [8] = Random-Forest-Classifier; Method [18] = Gradient-Boosting-Machine Classifier process.

## Conclusion & future scopes

This paper is one example that showcases an application of a holistic graph-based model that predicts the risk of GDM using advanced GraphSAGE, Relational Graph Convolutional Networks (R-GCN), Temporal Graph Attention Networks (TGAT), and other techniques including BioBERT pretraining, federated learning, and DropEdge regularization. The proposed model shows significant improvements over baseline machine learning techniques in all key performance metrics, that is, classification accuracy of 88.2%, AUC of 0.90, RMSE for the temporal prediction of 0.30, F1 score of 0.86, precision of 0.83, recall of 0.85, and attention agreement with domain experts of 82%. These results emphasize that this model is not only capable of accurate GDM outcome prediction but also can capture the complex, multi-dimensional relationships between clinical, demographic, and temporal factors that drive the risk of developing GDM. Baseline methods such as Method [5], Method [8], and Method [18] achieve significantly lower performance on these metrics. Method [5] reached classification accuracy of 76.5% with an AUC value of 0.75 and an RMSE of 0.45, while Method [8] performed moderately better at an accuracy of 80.1% and AUC equal to 0.80. Clinical relevance-the performance improvements of the proposed model especially at the level of predictive accuracy and temporal forecasting would hold clinical significance in early detection and management towards personalization in the handling of GDM to ultimately prevent complications such as macrosomia, preterm birth, and long-term metabolic issues in the processing. The proposed system works on heterogeneous data, wherein there are temporal dynamics; multi-relational factors arise, and the application considers privacy-preserving federated learning, making it dissimilar from conventional systems. Moreover, the explainability of the model, as shown by the high attention agreement with domain experts at 82%, increases the trustworthiness and clinical applicability of the model. Insights into the relative importance of various risk factors can be useful for targeted interventions by informed clinicians based on patient profiles. Although it consumes more computational resources and needs more training timestamp (5.5 h), such a trade-off in favor of superior performance is quite justifiable, especially considering the fact that, once trained, it promises to provide accurate predictions without demanding the necessity of frequent retraining. And that is why it will be pretty attractive in real-time clinical settings where decision support systems should, at the same time, both be accurate and interpretable in process.

We found that the graph-based framework showed better discrimination/temporal modeling/interpretability within both the internally-tested pregnancy cohort/public feature-limited benchmark environment. Our results suggest that relational/temporal modeling can help earlier GDM risk-stratification; nonetheless, clinical generalizability of the framework needs further confirmation through independent/prospectively-collected multi-center GDM cohorts before routine applications.

There are many areas for furthering the model, and enhancing its performance, so it can be practically applicable. These include introducing new sources of data, such as markers that would provide a wealthier representation of the patients’ risk profiles. Adding even larger and more general datasets, perhaps coming from several different geographical locations and ethnic groups, would definitely enhance generalizability to an even greater extent. Future work in terms of temporal analysis may include further refinement of the TGAT model to better handle more challenging cases of irregular time-series data, such as varying glucose measurement frequencies or missing values at pregnancy. Also, the federated learning protocol can be extended in order to provide better privacy guarantees for training on decentralized data so as to pave the way for further wide-scale deployment in real-world healthcare settings. The current model does not provide for updating patient embeddings in real-time with new data arrival; inclusion of such adaptive learning techniques will enhance predictive accuracy in dynamic clinical settings. Lastly, since personalized medicine makes more and more specific requests, involving multi-task learning frameworks will add more relevant knowledge about the forecasting of complications development for GDM; therefore, the system has wider implications in health care for multiple directions of decision Making. Generally speaking, the new graph-based approach is really novel at this point, promising not only superior performance but satisfactory interpretability at different temporal instance sets. This model is the potential harbinger of change in the care for GDM and can provide personalized risk management and, as such, improve the earlier detection and management process. Therefore, in the near future, scalability, adaptability, and integration into clinical workflows could focus on work to improve outcomes for health in mothers and newborns for different clinical scenarios.

### Expert agreement and explainability evaluation

Expert agreement was assessed by way of a formal review process as opposed to merely visually reviewing illustrative cases. Each selected test-case received a ranked-list of the top 10 contributing risk-factors (based upon attention-weights and post-hoc attribution-scores) produced by the model. Three domain-experts with expertise in obstetrics, endocrinology, and maternal-metabolic risk-assessment independently reviewed these lists. All reviewers were blinded to the identity of the model producing the rankings as well as the predicted-probability categories associated with those rankings. Consensus was defined as having at least two out of three reviewers agree that a ranked-factor was clinically-plausible for the corresponding-patient-profile. Prior to aggregating reviewer-consensus across patients in the test-set, inter-rater-agreement was measured utilizing Fleiss’ kappa statistic. Finally, expert-agreement-percentage was calculated based upon all held-out test-subset-patients rather than illustrative examples. Explainability evaluation was tied directly to predictions at the individual-patient level produced by the model from the test-split. For each patient, the ranked-factors were compared against clinical variables available prior to the label assignment—including glucose related-measures, BMI, maternal-age, obstetric-history, blood-pressure Indicators, family-history and longitudinal-trimester-trends wherever possible. As such, attention-agreement represented clinically Interpretable model-behavior on evaluated predictions as opposed to manually-prepared example-demonstrations.

### Limitations

This study has several limitations which restrict how one might interpret generalizability. First, since the UCI Pima Indians Diabetes dataset is not a gestational-diabetes cohort it was used exclusively as a limited-feature public diabetes benchmark as opposed to an externally Validating GDM dataset. Second, since the complete multimodal primary cohort referred to in methods cannot be openly distributed because it includes institutionally-controlled clinical data; longitudinally-recorded pregnancy data; and text representation-derived features—we cannot make it publicly accessible. Third, although internal-patient-level splits minimize leakage and facilitate controlled comparisons among others—our current evidence does not demonstrate sufficient validation to assume generalizability to previously unseen hospital/population/diagnostic workflow/pregnancy care settings. Thusly, we believe that future validations should employ prospectively-collected independent GDM-cohorts with harmonized definitions for outcomes/subgroup-reporting/site-specific and pre-registration of model-evaluation protocols.

## Data Availability

Only reproducing the publicly-accessible links in the paper allow for reproducing the publicly-accessible benchmark experiments and not reproduceable for the rest of the multimodal primary cohort used in generating the graph-based GDM analysis. The primary dataset represents privacy sensitive clinical data, longitudinally recorded structured EHR data, trimester aligned clinical measures, and text representation-derived features that cannot be shared due to data-use restrictions instituted by institutions and patient confidentially requirements. Aggregate de Identified statistics, dictionaries of features, processing parameters, model configuration files and script for benchmarks may be shared by the authors upon reasonable requests subject to approval of their respective institutions. The UCI Pima Indians Diabetes dataset is only being used as a feature-limited public diabetes benchmark and should not be assumed as an external validation cohort for GDM. The data sets used and/or analysed during the current study are available https://www.kaggle.com/datasets/uciml/pima Indians-diabetes-database https://www.kaggle.com/datasets/sumathisanthosh/gestational-diabetes-mellitus-gdm-data-set

## Abbreviations

GDM: Gestational diabetes mellitus
IoT: Internet of things
PSO: Particle swarm optimization
RNA: Ribonucleic acid
SVM: Support vector machine
LSTM: Long short-term memory
DVT: Deep vein thrombosis
BMI: Body mass index
T2DM: Type 2 diabetes mellitus
FPG: Fasting plasma glucose
HOMA IR: Homeostasis model assessment insulin resistance
AI: Artificial intelligence
KNN: K-nearest neighbors
GDM: Gestational diabetes mellitus
SNP: Single nucleotide polymorphism
CV: Cross validation
RF: Random forest
ANN: Artificial neural network
ROC: Receiver operating characteristic
AUC: Area under the curve
HR: Heart rate
F1: F1 Score
RMSE: Root mean square error
CRP: C-Reactive protein
HbA1c: Hemoglobin A1c
LASSO: Least absolute shrinkage and selection operator
SVM: Support vector machine
ROC: Receiver operating characteristic
MLP: Multilayer perceptron
LDA: Linear discriminant analysis
PCA: Principal component analysis
NN: Neural network
RNN: Recurrent neural network
SIFT: Scale invariant feature transform
K-Fold: K-fold cross validation
SGD: Stochastic gradient descent
TPR: True positive rate
FNR: False negative rate
TNR: True negative rate
FPR: False positive rate
GNN: Graph neural network
CNN: Convolutional neural network
DNN: Deep neural network
MCMC: Markov Chain Monte Carlo
UCI: University of California Irvine
ROC-AUC: Receiver operating characteristic—area under curve
T2DM: Type 2 diabetes mellitus

## References

[1] Moreno, V. A. et al. Exploring Beliefs, Concerns, Prenatal Care Advice, and Sources of Information About Gestational Weight Gain Among Immigrant Central American Pregnant Women in the United States. *Int. J. Environ. Res. Public Health* **21**(12), 1672. 10.3390/ijerph21121672 (2024). 39767510 10.3390/ijerph21121672 PMC11675826

[2] Gulersen, M. et al. Maternal Morbidity Associated with Early Preterm Birth in Low-Risk Singleton Pregnancies. *J. Clin. Med.* **13**(23), 7061. 10.3390/jcm13237061 (2024). 39685519 10.3390/jcm13237061 PMC11642920

[3] Zampieri, G. et al. Contributions Regarding the Study of Pulsatility and Resistivity Indices of Uterine Arteries in Term Pregnancies—A Prospective Study in Bucharest, Romania. *Diagnostics.* **14**(22), 2556. 10.3390/diagnostics14222556 (2024). 39594222 10.3390/diagnostics14222556 PMC11593153

[4] Genova, M. P., Ivanova, I., Naseva, E. & Atanasova, B. Association Between Zinc Status and Insulin Resistance/Sensitivity Check Indexes in Gestational Diabetes Mellitus. *Int. J. Mol. Sci.* **25**(22), 12193. 10.3390/ijms252212193 (2024). 39596259 10.3390/ijms252212193 PMC11594766

[5] Siemers, K. M., Joss Moore, L. A. & Baack, M. L. Gestational Diabetes-like Fuels Impair Mitochondrial Function and Long-Chain Fatty Acid Uptake in Human Trophoblasts. *Int. J. Mol. Sci.* **25**(21), 11534. 10.3390/ijms252111534 (2024). 39519087 10.3390/ijms252111534 PMC11546831

[6] Payot, M. D., Villavieja, A. & Pineda-Cortel, M. R. Preliminary Investigation of Potential Early Biomarkers for Gestational Diabetes Mellitus: Insights from *PTRPG* and *IGKV2D-28* Expression Analysis. *Int. J. Mol. Sci.* **25**(19), 10527. 10.3390/ijms251910527 (2024). 39408856 10.3390/ijms251910527 PMC11476507

[7] Seifert, C. T. et al. Galectin-7 Expression in the Placentas of Women with Gestational Diabetes Mellitus. *Int. J. Mol. Sci.* **25**(18), 10186. 10.3390/ijms251810186 (2024). 39337670 10.3390/ijms251810186 PMC11432196

[8] Rüegg, L., Vonzun, L., Zepf, J., Strübing, N., Möhrlen, U., Mazzone, L., Meuli, M., Spina Bifida Study Group, Ochsenbein-Kölble N. Gestational Diabetes in Women with Fetal Spina Bifida Repair—Influence of Perioperative Management. *Journal of Clinical Medicine*. 13(17):5029. 10.3390/jcm13175029. (2024). 10.3390/jcm13175029 PMC11395906 39274242

[9] Hung, S.-C. et al. Lysophosphatidylcholine Impairs the Mitochondria Homeostasis Leading to Trophoblast Dysfunction in Gestational Diabetes Mellitus. *Antioxidants.* **13**(8), 1007. 10.3390/antiox13081007 (2024). 39199251 10.3390/antiox13081007 PMC11351454

[10] Filip, C. et al. The Burden of Deep Vein Thrombosis and Risk Factors in Pregnancy and Postpartum—Mirroring Our Region’s Particularities. *J. Clin. Med.* **13**(16), 4705. 10.3390/jcm13164705 (2024). 39200848 10.3390/jcm13164705 PMC11355405

[11] Mandić Marković, V. et al. Biochemical Markers in the Prediction of Pregnancy Outcome in Gestational Diabetes Mellitus. *Medicina* **60**(8), 1250. 10.3390/medicina60081250 (2024). 39202531 10.3390/medicina60081250 PMC11356194

[12] Wang, Z. et al. Association of Beverage Consumption during Pregnancy with Adverse Maternal and Offspring Outcomes. *Nutrients* **16**(15), 2412. 10.3390/nu16152412 (2024). 39125293 10.3390/nu16152412 PMC11314345

[13] Lu, H. Y. et al. Digital Health and Machine Learning Technologies for Blood Glucose Monitoring and Management of Gestational Diabetes. *IEEE Rev. Biomed. Eng.* **17**, 98–117. 10.1109/RBME.2023.3242261 (2024). 37022834 10.1109/RBME.2023.3242261 PMC7615520

[14] Gomes Filho, E., Pinheiro, P. R., Pinheiro, M. C. D., Nunes, L. C. and Gomes, L. B. G. Heterogeneous Methodology to Support the Early Diagnosis of Gestational Diabetes, In: IEEE Access, vol. 7, pp. 67190–67199, 10.1109/ACCESS.2019.2903691. (2019).

[15] Kolozali, Ş, White, S. L., Norris, S., Fasli, M. & van Heerden, A. Explainable Early Prediction of Gestational Diabetes Biomarkers by Combining Medical Background and Wearable Devices: A Pilot Study With a Cohort Group in South Africa. *IEEE J. Biomed. Health Inform.* **28**(4), 1860–1871. 10.1109/JBHI.2024.3361505 (2024). 38345955 10.1109/JBHI.2024.3361505

[16] Marzouk, R., Alluhaidan, A. S. & ElRahman, S. A. An Analytical Predictive Models and Secure Web-Based Personalized Diabetes Monitoring System. *IEEE Access* **10**, 105657–105673. 10.1109/ACCESS.2022.3211264 (2022).

[17] Khan, M. Z., Mangayarkarasi, R., Vanmathi, C. & Angulakshmi, M. Bio Inspired PSO for Improving Neural Based Diabetes Prediction System. *Journal of ICT Standardization* **10**(2), 179–199. 10.13052/jicts2245-800X.1025 (2022).

[18] Saleh Al Reshan, M. et al. An innovative ensemble deep learning clinical decision support system for diabetes prediction, In: IEEE Access, vol. 12, pp. 106193–106210, 10.1109/ACCESS.2024.3436641. (2024).

[19] Ali Linkon, A. et al. Evaluation of feature transformation and machine learning models on early detection of diabetes mellitus, In: IEEE Access, vol. 12, pp. 165425–165440, 10.1109/ACCESS.2024.3488743. (2024).

[20] Alnowaiser, K. Improving Healthcare Prediction of Diabetic Patients Using KNN Imputed Features and Tri-Ensemble Model. *IEEE Access* **12**, 16783–16793. 10.1109/ACCESS.2024.3359760 (2024).

[21] Zhao, Y. et al. Hypertension risk prediction models for patients with diabetes based on machine learning approaches. *Multimed Tools Appl* **83**, 59085–59102. 10.1007/s11042-023-17926-x (2024).

[22] Kurt, B. et al. Prediction of gestational diabetes using deep learning and Bayesian optimization and traditional machine learning techniques. *Med Biol Eng Comput* **61**, 1649–1660. 10.1007/s11517-023-02800-7 (2023). 36848010 10.1007/s11517-023-02800-7 PMC9969040

[23] Ashisha, G. R. et al. Random Oversampling-Based Diabetes Classification via Machine Learning Algorithms. *Int J Comput Intell Syst* **17**, 270. 10.1007/s44196-024-00678-3 (2024).

[24] Feng, X., Cai, Y. & Xin, R. Optimizing diabetes classification with a machine learning-based framework. *BMC Bioinformatics* **24**, 428. 10.1186/s12859-023-05467-x (2023). 37957549 10.1186/s12859-023-05467-x PMC10644638

[25] Uddin, M. A. et al. Machine Learning Based Diabetes Detection Model for False Negative Reduction. *Biomedical Materials & Devices* **2**, 427–443. 10.1007/s44174-023-00104-w (2024).

[26] Du, J. et al. Visualization obesity risk prediction system based on machine learning. *Sci Rep* **14**, 22424. 10.1038/s41598-024-73826-6 (2024). 39342032 10.1038/s41598-024-73826-6 PMC11439005

[27] Darsareh, F. et al. Application of machine learning to identify risk factors of birth asphyxia. *BMC Pregnancy Childbirth* **23**, 156. 10.1186/s12884-023-05486-9 (2023). 36890453 10.1186/s12884-023-05486-9 PMC9993370

[28] Wu, Y. et al. Early prediction of gestational diabetes mellitus using maternal demographic and clinical risk factors. *BMC Res Notes* **17**, 105. 10.1186/s13104-024-06758-z (2024). 38622619 10.1186/s13104-024-06758-z PMC11021008

[29] Bai, X. et al. Development and evaluation of machine learning models for predicting large-for-gestational-age newborns in women exposed to radiation prior to pregnancy. *BMC Med Inform Decis Mak* **24**, 174. 10.1186/s12911-024-02556-6 (2024). 38902714 10.1186/s12911-024-02556-6 PMC11188254

[30] Khadidos, A. O. et al. Ensemble machine learning framework for predicting maternal health risk during pregnancy. *Sci Rep* **14**, 21483. 10.1038/s41598-024-71934-x (2024). 39277644 10.1038/s41598-024-71934-x PMC11401887

[31] Du, M. et al. Maternal Height Is an Independent Risk of Adverse Outcomes in Women with Gestational Diabetes Mellitus. *Diabetes Ther* **15**, 461–472. 10.1007/s13300-023-01512-3 (2024). 38104305 10.1007/s13300-023-01512-3 PMC10838893

[32] Soria-Contreras, D. C. et al. Lifetime history of gestational diabetes and cognitive function in parous women in midlife. *Diabetologia*10.1007/s00125-024-06270-w (2024). 39240352 10.1007/s00125-024-06270-w PMC11960863

[33] Yang, S. W. et al. Machine learning analysis with population data for prepregnancy and perinatal risk factors for the neurodevelopmental delay of offspring. *Sci Rep* **14**, 13993. 10.1038/s41598-024-64590-8 (2024). 38886474 10.1038/s41598-024-64590-8 PMC11183197

[34] Doğru, A., Buyrukoğlu, S. & Arı, M. A hybrid super ensemble learning model for the early-stage prediction of diabetes risk. *Med Biol Eng Comput* **61**, 785–797. 10.1007/s11517-022-02749-z (2023). 36602674 10.1007/s11517-022-02749-z

[35] Xu, Y. et al. Dietary inflammatory index as a predictor of prediabetes in women with previous gestational diabetes mellitus. *Diabetol Metab Syndr* **16**, 265. 10.1186/s13098-024-01486-7 (2024). 39506813 10.1186/s13098-024-01486-7 PMC11542452

[36] Rustam, F. et al. Enhanced detection of diabetes mellitus using novel ensemble feature engineering approach and machine learning model. *Sci Rep* **14**, 23274. 10.1038/s41598-024-74357-w (2024). 39375469 10.1038/s41598-024-74357-w PMC11458802

[37] Ordoñez-Guillen, N. E. et al. Machine learning based study for the classification of Type 2 diabetes mellitus subtypes. *BioData Mining* **16**, 24. 10.1186/s13040-023-00340-2 (2023). 37608329 10.1186/s13040-023-00340-2 PMC10463725

[38] Salvatori, B. et al. Identification and validation of gestational diabetes subgroups by data-driven cluster analysis. *Diabetologia* **67**, 1552–1566. 10.1007/s00125-024-06184-7 (2024). 38801521 10.1007/s00125-024-06184-7 PMC11343786

[39] Naito, T. et al. Machine learning reveals heterogeneous associations between environmental factors and cardiometabolic diseases across polygenic risk scores. *Commun Med* **4**, 181. 10.1038/s43856-024-00596-7 (2024). 39304733 10.1038/s43856-024-00596-7 PMC11415376

[40] Belaghi, R. A. Prediction of preterm birth in multiparous women using logistic regression and machine learning approaches. *Sci Rep* **14**, 21967. 10.1038/s41598-024-60097-4 (2024). 39304672 10.1038/s41598-024-60097-4 PMC11415355

[41] Solomon, D.D., Sonia, Kumar, K. et al. Extensive Review on the Role of Machine Learning for Multifactorial Genetic Disorders Prediction. *Arch Computat Methods Eng* **31**, 623–640. 10.1007/s11831-023-09996-9. (2024).

[42] Long, W. et al. Development of a predictive model for the risk of microalbuminuria: comparison of 2 machine learning algorithms. *J Diabetes Metab Disord* **23**, 1899–1908. 10.1007/s40200-024-01440-4 (2024). 39610509 10.1007/s40200-024-01440-4 PMC11599703

[43] Aghajanian, S. et al. Prediction of post-delivery hemoglobin levels with machine learning algorithms. *Sci Rep* **14**, 13953. 10.1038/s41598-024-64278-z (2024). 38886458 10.1038/s41598-024-64278-z PMC11183065

[44] Kokori, E. et al. The role of machine learning algorithms in detection of gestational diabetes; a narrative review of current evidence. *Clin Diabetes Endocrinol* **10**, 18. 10.1186/s40842-024-00176-7 (2024). 38915129 10.1186/s40842-024-00176-7 PMC11197257

[45] Kaya, Y. et al. The early prediction of gestational diabetes mellitus by machine learning models. *BMC Pregnancy Childbirth* **24**, 574. 10.1186/s12884-024-06783-7 (2024). 39217284 10.1186/s12884-024-06783-7 PMC11365266

[46] Kang, B. S. et al. Prediction of gestational diabetes mellitus in Asian women using machine learning algorithms. *Sci Rep* **13**, 13356. 10.1038/s41598-023-39680-8 (2023). 37587201 10.1038/s41598-023-39680-8 PMC10432552

[47] Cubillos, G. et al. Development of machine learning models to predict gestational diabetes risk in the first half of pregnancy. *BMC Pregnancy Childbirth* **23**, 469. 10.1186/s12884-023-05766-4 (2023). 37353749 10.1186/s12884-023-05766-4 PMC10288662

[48] Chan, Y. N. et al. A machine learning approach for early prediction of gestational diabetes mellitus using elemental contents in fingernails. *Sci Rep* **13**, 4184. 10.1038/s41598-023-31270-y (2023). 36918683 10.1038/s41598-023-31270-y PMC10015050

[49] Li, Yx., Liu, Yc., Wang, M. et al. Prediction of gestational diabetes mellitus at the first trimester: machine-learning algorithms. *Arch Gynecol Obstet* **309**, 2557–2566. 10.1007/s00404-023-07131-4. (2024). 10.1007/s00404-023-07131-4 37477677

[50] Nuthakki, P. & Kumar, T. P. Machine learning-based early detection of diabetes risk factors for improved health management. *Multimed Tools Appl*10.1007/s11042-024-18728-5 (2024).

